# The *comER* Gene Plays an Important Role in Biofilm Formation and Sporulation in both *Bacillus subtilis* and *Bacillus cereus*

**DOI:** 10.3389/fmicb.2016.01025

**Published:** 2016-06-28

**Authors:** Fang Yan, Yiyang Yu, Luyao Wang, Yuming Luo, Jian-hua Guo, Yunrong Chai

**Affiliations:** ^1^Department of Plant Pathology, Nanjing Agricultural University, NanjingChina; ^2^Department of Biology, Northeastern University, Boston, MAUSA; ^3^Jiangsu Collaborative Center of Regional Modern Agriculture and Environmental Protection, NanjingChina; ^4^Engineering Center of Bioresource Pesticide in Jiangsu Province, Key Laboratory of Integrated Management of Crop Diseases and Pests, NanjingChina

**Keywords:** ComER, biofilm, Sda, *Bacillus subtilis*, *Bacillus cereus*

## Abstract

Bacteria adopt alternative cell fates during development. In *Bacillus subtilis*, the transition from planktonic growth to biofilm formation and sporulation is controlled by a complex regulatory circuit, in which the most important event is activation of Spo0A, a transcription factor and a master regulator for genes involved in both biofilm formation and sporulation. In *B. cereus*, the regulatory pathway controlling biofilm formation and cell differentiation is much less clear. In this study, we show that a novel gene, *comER*, plays a significant role in biofilm formation as well as sporulation in both *B. subtilis* and *B. cereus*. Mutations in the *comER* gene result in defects in biofilm formation and a delay in spore formation in the two *Bacillus* species. Our evidence supports the idea that *comER* may be part of the regulatory circuit that controls Spo0A activation. *comER* likely acts upstream of *sda*, a gene encoding a small checkpoint protein for both sporulation and biofilm formation, by blocking the phosphor-relay and thereby Spo0A activation. In summary, our studies outlined a conserved, positive role for *comER*, a gene whose function was previously uncharacterized, in the regulation of biofilm formation and sporulation in the two *Bacillus* species.

## Introduction

*Bacillus subtilis* and *B. cereus* are closely related, soil-dwelling spore-forming bacteria. In the environment, both species are found in the rhizosphere and both are considered as biological control agents that help plants fend off infections caused by plant pathogens and sometimes even fungi and parasites (Emmert and Handelsman, 1999; Berg et al., 2005; Aliye et al., 2008). Therefore they have drawn great interest in the agricultural field. In both *B. subtilis* and *B. cereus*, it is proposed that the biological control activities in part have to do with their ability to form multicellular communities, or biofilms, on the root surface of the plants (Bais et al., 2004; Chen et al., 2012, 2013; Beauregard et al., 2013). Studies show that wild-type (WT) strains of *B. subtilis* capable of forming robust biofilms have a much higher efficacy in the biological control activity than the mutants deficient in biofilm formation (Chen et al., 2013). For *B. cereus*, aside from being a biological control agent, some strains are also known to cause foodborne illness or even more severe diseases such as endophthalmitis and meningitis (Kotiranta et al., 2000). The pathogenesis of *B. cereus* is related to several enterotoxins and hemolysins produced by some *B. cereus* strains, such as hemolysin BL (Hbl), non-hemolytic enterotoxin (Nhe), and cytotoxin K (CytK; Gohar et al., 2008).

**FIGURE 1:**
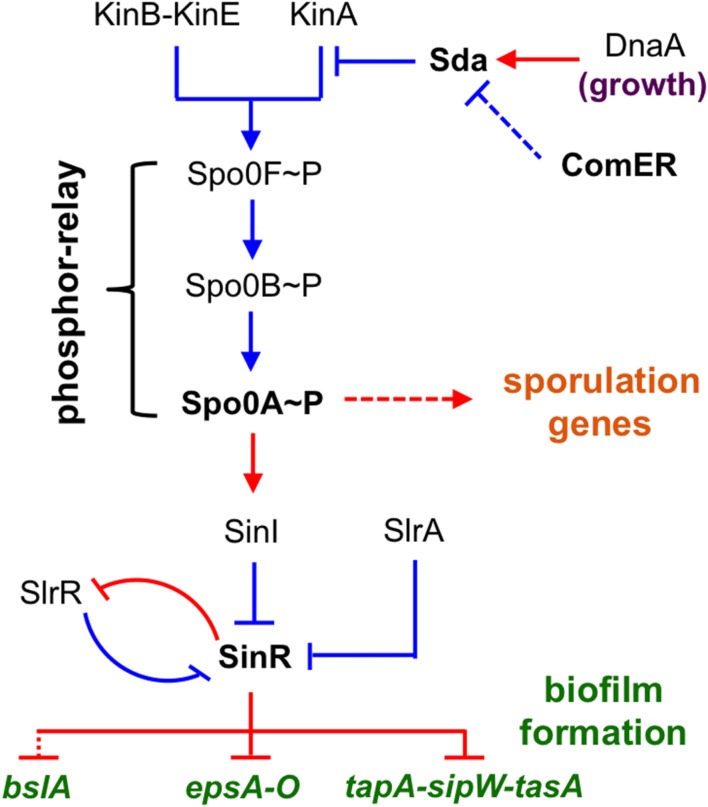
**A schematic presentation of the regulatory circuit for the control of alternative cell fates in *B. subtilis*.** Spo0A is positioned at the center of the regulatory circuit, controlling genes involved in both sporulation and biofilm formation. Spo0A is activated by protein phosphorylation (Spo0A~P), often through a phosphor-relay (initiated from multiple Kin kinases and mediated by Spo0F and Spo0B). Sda is a checkpoint protein that blocks the phosphor-relay from KinA to Spo0F and thus Spo0A activation during cell exponential growth. *sda* is activated by DnaA during exponential growth. SinR is the biofilm master repressor for the matrix genes *tapA-sipW-tasA*, *espA-O*, and *bslA*. SinR is counteracted by two parallel anti-repressors (SinI and SlrA) during biofilm induction (Kearns et al., 2005; Kobayashi, 2008; Chai et al., 2009). SlrR is another counteracting protein of SinR and shares strong amino acid sequence similarity with SinR (Chu et al., 2008). These two proteins constitute a self-reinforcing double-negative loop for the mutually exclusive control of matrix genes and free-living genes (Chu et al., 2008). Red, gene regulation; blue, protein–protein interaction.

In *B. subtilis*, the genetic circuitry that controls biofilm formation has been well characterized (Aguilar et al., 2007; Shank and Kolter, 2011; Vlamakis et al., 2013). Multiple histidine kinases (KinA, KinB, KinC, KinD, and KinE) sense various environmental and physiological signals and collectively act, either directly on the master regulator Spo0A through protein phosphorylation, or indirectly via a phosphor-relay (mediated by the phospho-transfer proteins Spo0F and Spo0B; **Figure 1**; Burbulys et al., 1991; Jiang et al., 2000; McLoon et al., 2011b). Spo0A functions as a master regulator for endospore formation by controlling hundreds of genes involved in the sporulation process in *B. subtilis* (Molle et al., 2003; Fujita et al., 2005). Spo0A also regulates biofilm formation by activating a small gene *sinI*, which encodes an anti-repressor for the biofilm master repressor SinR (**Figure 1**) (Bai et al., 1993; Kearns et al., 2005; Chai et al., 2011; Newman et al., 2013). SinR directly represses two operons, *tapA-sipW-tasA* and *epsA-O*, that are responsible for making the protein fibers (TasA) and exopolysaccharides (EPS) of the biofilm matrix, respectively (**Figure 1**) (Kearns et al., 2005; Chu et al., 2006). Recent studies suggest that the biofilm matrix of *B. subtilis* also consists of a small hydrophobin BslA (Hobley et al., 2013). The gene for BslA was shown to be under the control of the response regulator DegU and the transcription repressors, SinR and AbrB, either directly or indirectly (Verhamme et al., 2009). The biofilm repressor SinR also represses the gene for an additional regulatory protein SlrR (Chu et al., 2008; Kobayashi, 2008), which shares strong amino acid sequence similarity with SinR (Chu et al., 2008). Evidence indicates that SinR and SlrR constitute a self-reinforcing double-negative loop that locks cells in the matrix-producing state (**Figure 1**) (Chai et al., 2010). A third small antagonist of SinR, SlrA, was also shown to directly interact with SinR and relieve SinR-mediated repression (**Figure 1**) (Chai et al., 2009; Newman and Lewis, 2013). Molecular details of how SinR interacts SinI, SlrR, and SlrA were further characterized by recent studies using structural and biochemical approaches (Newman and Lewis, 2013; Newman et al., 2013).

In the genetic network for the control of alternative cell fates in *B. subtilis* (planktonic growth, biofilm formation, sporulation, etc.), Spo0A is positioned at the center of the network (**Figure 1**). A *spo0A* null mutant is severely defective in both sporulation and biofilm formation (Branda et al., 2001, 2004; Hamon and Lazazzera, 2001). Activation of Spo0A does not simply rely on protein phosphorylation, but is under the control of complex regulations (Ireton et al., 1993; Perego et al., 1994; Jiang et al., 2000). For instance, the activity of Spo0A is counter-regulated by protein dephosphorylation by multiple phosphatases (Perego et al., 1994). Spo0A activation is also reinforced by a positive feedback mechanism, in which the expression of several genes involved in the phospho-relay (such as *spo0F* and *spo0B*) is further activated by Spo0A (Fujita and Losick, 2005; Chastanet et al., 2010). Lastly, Spo0A activity is also controlled by Sda, a small checkpoint protein for sporulation by blocking the phospho-transfer from the sensory histidine kinase A (KinA) to the intermediate phosphor carrier Spo0F, thereby blocking or delaying Spo0A activation (**Figure 1**) (Burkholder et al., 2001; Whitten et al., 2007).

*Bacillus cereus* has also been reported to be capable of forming submerged or surface-attached biofilms under laboratory conditions as well as on the surface of plant roots (Emmert and Handelsman, 1999; Chandramohan et al., 2009; Shemesh and Chai, 2013; Gao et al., 2015). In contrast to *B. subtilis*, few genes involved in biofilm formation have been characterized in *B. cereus* and the regulatory mechanisms that control biofilm formation are poorly understood (Lindbäck et al., 2012; Caro-Astorga et al., 2015; Gao et al., 2015). One recent study suggested that the homologous gene to *spo0A* of *B. subtilis* is important for biofilm formation in *B. cereus* (Gao et al., 2015). Another study showed that genes homologous to *sipW* and *tasA* of *B. subtilis* also seem to be important for production of adhesion-like fibers for the biofilm matrix in *B. cereus* (Caro-Astorga et al., 2015). A global regulator CodY for cell stationary phase growth was also shown to be important for biofilm formation in *B. cereus* (Lindbäck et al., 2012). However, even with the recent progresses, current knowledge about *B. cereus* biofilm formation is still largely lacking.

**FIGURE 2:**
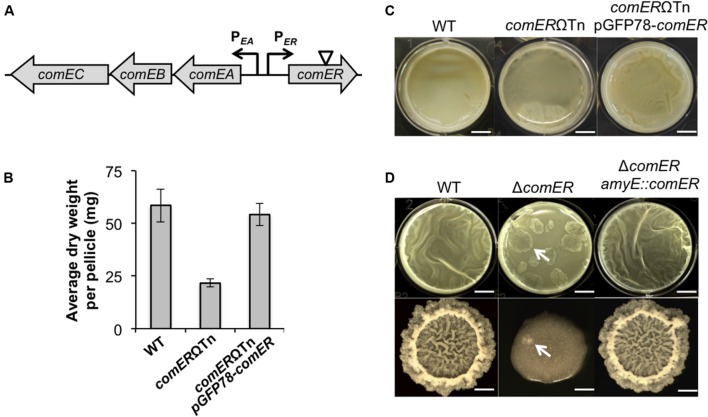
***comER* is important for biofilm formation in both *B. subtilis* and *B. cereus.***(A)** A schematic drawing of the chromosomal region in *B. subtilis* containing divergently transcribed *comER* and the *comEA-EB-EC* operon (indicated by arrows).** *comEA* and *comEC* encode structural proteins involved in DNA uptake during genetic competence (Hahn et al., 1993). The role of *comEB* is unclear and the gene is dispensable for genetic competence (Hahn et al., 1993). The position of the mini-Tn10 transposon insertion in the *comER* gene on the chromosome of *B. cereus* AR156 is indicated by the triangle. **(B)** Pellicle biofilm formation by the wild type (WT) (AR156) and the *comER* mutant (B168), and the *comER* complementation strain (YY298) of *B. cereus*. Scale bars, 4 mm. **(C)** Quantitative analysis of the biomass of pellicle biofilms from the WT (AR156), the *comER* transposon insertion mutant (B168), and the *comER* complementation strain (YY298) of *B. cereus*. Values in y-axis represent average dry weight (mg) per pellicle. Assays were done in triplicate. **(D)** Pellicle and colony biofilm formation by the WT (3610), the *comER* mutant (B165), and the *comER* complementation strain (YL46) in *B. subtilis*. Scale bars in the upper panels (pellicles) represent 4 mm in length and those in the lower panels (colonies) represent 3 mm in length. Arrows point to putative suppressors of *B. subtilis* Δ*comER* emerged during both pellicle and colony biofilm development.

We aimed to identify genes that are important for biofilm formation in *B. cereus* and further characterize the function of those genes. In our study, we used an environmental isolate of *B. cereus* (AR156; Niu et al., 2011). AR156 is capable of forming thick floating pellicle biofilms under laboratory conditions (presented in this study) and shows strong biological control activities toward various plant pathogens (Niu et al., 2011). In a parallel study, we conducted a genome-wide random insertion mutagenesis in AR156 by using the mini-Tn10 based transposon system. A total of ~10,000 transposon insertion mutants were screened for alteration of the biofilm phenotype. About 23 such mutants were subsequently obtained (see section “Materials and Methods”). In this study, we focused on one such mutant that has a transposon insertion in the gene annotated as *comER* (**Figure 2A**). *comER* encodes a protein that resembles Δ^1^-pyrroline 5-carboxylate reductase, an enzyme involved in the last step of proline biosynthesis (Belitsky et al., 2001). However, previous evidence suggests that *comER* does not have any significant role in proline biosynthesis in *B. subtilis* (Inamine and Dubnau, 1995; Belitsky et al., 2001). Therefore, the exact function of *comER* remains unclear. In this work, we show that the *comER* gene plays an important role in biofilm formation and sporulation in both *B. cereus* and *B. subtilis*. Based on our evidence, we propose that *comER* may be part of the regulatory pathway involved in activation of Spo0A, the master regulator for biofilm formation and sporulation in the two *Bacillus* species.

## Materials and Methods

### Strains and Media

*Bacillus subtilis* and *B. cereus* strains were routinely cultured in Lysogenic broth (LB; 10 g tryptone, 5 g yeast extract, and 5 g NaCl per liter broth) at 37°C. All strains used in this study are described in Supplementary Table S1. All primers used in this study are listed in Supplementary Table S2. For assays of biofilm formation, two different biofilm media, LBGM and MSgg, were used. LBGM is composed of LB broth (or solidified LB agar) supplemented with 1% of glycerol and 100 μM MnSO_4_ for *B. subtilis* or 200 μM MnSO_4_ for *B. cereus* (Shemesh and Chai, 2013). The recipe for MSgg was described previously (Branda et al., 2001). Sporulation assays were performed in Difco Sporulation (DS) medium (Schaeffer et al., 1965; Nicholson and Setlow, 1990). Antibiotics were added at the following final concentrations: 100 μg/ml of spectinomycin, 5 μg/ml of chloramphenicol, 5 μg/ml of tetracycline, and 1 μg/ml of erythromycin plus 2.5 μg/ml of lincomycin (for selection of Mls resistance). Chemicals were purchased from Sigma. Restriction enzymes and other enzymes for molecular cloning were obtained from New England Biolabs. All primers were ordered from IDT DNA technology. DNA sequencing was performed at Genewiz.

### Transposon Mutagenesis

The plasmid pIC333 (Steinmetz and Richter, 1994) containing a mini-Tn10 element was used for random transposon insertion mutagenesis in *B. cereus* AR156. The pIC333 plasmid was first introduced into AR156 by electroporation, resulting in strain B79. Transposon mutagenesis was performed in B79 by following a similar protocol described in a previous publication with modifications (Kearns et al., 2004). To explain briefly, B79 cells were grown at the permissive temperature (25°C, pIC333 contains a temperature-sensitive replication origin) to mid-log phase in LB medium supplemented with both spectinomycin (100 μg/ml) and erythromycin (1 μg/ml). The culture was then diluted 1:100 into fresh LB medium supplemented with only spectinomycin (100 μg/ml) and the temperature was shifted from 25 to 45°C (non-permissive temperature for pIC333 replication) for overnight shaking growth of the bacterial cells. These two steps were repeated 8–10 times. At the end, appropriate dilutions of the cultures were plated on LB agar media supplemented with spectinomycin, and the plates were incubated at 45°C overnight. Individual transposon insertion mutants were picked, purified, and confirmed to be resistant to spectinomycin (Sp^R^) but sensitive to erythromycin (Mls^S^). Those transposon insertion mutants (Sp^R^, Mls^S^) were spotted on solid biofilm medium LBGM or inoculated into LBGM broth. Plates were incubated statically at 30°C for about three days for colony biofilm development or two days for pellicle biofilm development. Insertion mutants that showed altered biofilm morphology in either pellicle or colony biofilms were picked. The altered biofilm phenotype of the candidate mutants was verified in repeated biofilm assays. A total of ~10,000 transposon insertion mutants were initially screened for alteration of the biofilm phenotypes. About 23 such mutants were subsequently obtained.

Next, to map the transposon insertion sites on the chromosome in the transposon insertion mutants, genomic DNA was prepared from those mutants by using a commercial kit (Promega, USA). 5 μg of purified genomic DNA was digested with *Eco*RI or *Hind*III, purified, and ligated overnight at 16°C. The ligation mixture was transformed to *Escherichia coli* DH5α. Plasmid DNA was prepared from *E. coli* and sent for DNA sequencing by using the primers Tn10-113-98 and Tn10-2235-2249 listed in Supplementary Table S2. The two primers allow sequence reading outward from the border sequences of the transposon insertion sites. The obtained DNA sequences were used to map the transposon insertion sites by aligning the sequence with the genome sequences of both *B. cereus* ATCC14579 (Ivanova et al., 2003) and AR156 (GenBank Access Number CP015589).

### Strain Construction

The deletion mutation in the *comER* or *sda* gene in *B. subtilis* NCIB3610 (hereafter designated as 3610) was generated by long flanking PCR mutagenesis (Wach, 1996). The four primers (delta-comER-P1 to delta-comER-P4) used for *comER* mutagenesis are listed in Supplementary Table S2. A deletion mutation in *sda* (Tet^R^) in 3610 was constructed similarly by using the primers of delta-sda-P1 to delta-sda-P4. The *comER* insertion mutant of *B. cereus* AR156 was obtained from mini-Tn10 transposon insertion mutagenesis as described above. To construct the complementation strain of Δ*comER* in *B. subtilis* 3610, the promoter and the coding sequences of *comER* were amplified by PCR using the primers P*_comER_*-F1 and P*_comER_*-R2. The PCR product was then cloned into the vector pDG1662 (Guérout-Fleury et al., 1996) between the *Eco*RI and *Bam*HI sites. The recombinant plasmid was first introduced into PY79 by transformation for integration at the *amyE* locus by double crossover recombination, and then to 3610 derivatives by SPP1 phage mediated general transduction. To construct the deletion mutation in the *yqeK* or *proH* genes, or to construct the strain with the P*_abrB_*-*lacZ* or *sdpC*::*sdpC*-*lacZ* reporter fusions, genomic DNA containing the corresponding deletion mutation or the promoter fusion was prepared from the derivative strain of PY79 or 168 (listed in Supplementary Table S1) and was introduced into 3610 or 3610 derivatives by either genetic transformation or by SPP1-mediated general transduction according to the published protocols (Yasbin and Young, 1974; Kearns et al., 2005).

To construct the *comER* complementation strain in *B. cereus* AR156, the *comER* gene was PCR amplified by using primers Bc-comER-OE-F and Bc-comER-OE-R (Supplementary Table S2) and AR156 genomic DNA. The PCR product was doubly digested by XbaI and HindIII, and then cloned into the pGFP78 plasmid (also digested by XbaI and HindIII; Gao et al., 2015). The recombinant plasmid (pGFP78-*comER*) was introduced into the *comER* insertional mutant of *B. cereus* (B168) by electroporation. Electroporation was carried out in a 0.2 cm cuvette with a voltage selection of 1.2 kV for 3.1 ms. Aliquots were spread onto LB plates supplemented with appropriate antibiotics. The *sdpC-gfp* reporter strains YY288 and YY289 were constructed by introducing the DNA fragment containing the *sdpC-gfp* reporter from *B. subtilis* train EG443 (Gonzalez-Pastor et al., 2003) to 3610 and B165 by SSP1 phage transduction.

### Bacterial Growth Curve

To compare the generation time of the WT and the mutant strains, cells were grown in LB medium to mid-log phase and then transferred to 25 ml of LBGM or the defined minimal medium MSgg (Branda et al., 2001) with a starting OD_600_ of 0.005. Cells continued to grow with shaking (250 rpm) at 37°C. Cell samples were collected every hour and OD_600_ of the cultures was measured by using the Bio-Rad Smartspec 3000.

### Analysis of Biofilm Formation

To analyze pellicle biofilm formation, cells were first grown in 3 ml LB broth to late exponential growth phase (OD_600_ = 1). Three microliters of culture was added to 3 ml of LBGM medium (a 1000-fold dilution) in 6-well or 12-well polyvinyl plates (VWR). The plates were incubated statically at 30°C for 24–48 h. For colony formation, 2 μl of the cells were spotted onto LBGM medium solidified with 1.5% agar. Plates were incubated at 30°C for 48–72 h prior to analysis. Images were taken by a Nikon CoolPix camera.

### Pellicle Dry Weight Assay

This assay was modified from a method originally developed in *B. subtilis* by Beauregard et al. (2013) to measure pellicle biofilm robustness. To perform the assay, pellicle biofilm formation was carried out in Costar 6-well polystyrene plates filled with Netwelf Insert with a polyester mesh bottom (opening size 440 μM; Corning). Biofilm media and *B. cereus* cells were added, and pellicles were allowed to develop for 48 h at 30°C. Individual wells were then removed and dried. Dried pellicles were carefully removed out of the well and weighed using an analytic balance. Assays were done in triplicate.

### Characterization of the Suppressor Mutants

The Δ*comER* deletion mutant of *B. subtilis* (B165) was inoculated in LBGM broth for pellicle biofilm development. Putative suppressors with more robust biofilm phenotypes occasionally emerged and were thus picked. These putative suppressors were streaked out on fresh LB plates and isolated as pure colonies. The robust biofilm phenotype of the suppressor mutants was repeatedly confirmed. Next, to identify the suppressor mutations, we applied a candidate approach by sequencing the selected genetic loci, including *sinR*, *abrB*, and *sda*.

Genomic DNA was prepared from 11 selected suppressor mutants by using the commercially available kit (Promega). The coding region of the *sinR*, *abrB*, and *sda* genes were PCR amplified by using primers listed in Supplementary Table S2. The PCR products were applied for DNA sequencing to search for putative mutations. In nine out of the 11 selected suppressor mutants, a mutation was identified in the coding region of *sinR* (Supplementary Figure S1).

### Assays of the Sporulation Efficiency

Heat kill experiments were performed to test the sporulation efficiency of the WT and the mutant strain of both *B. cereus* and *B. subtilis*. After being grown in DS medium for 24 h (for *B. subtilis*) or 36 h (for *B. cereus*), cell samples were serially diluted and plated on DS agar media to determine the number of total cells by counting the number of colonies on the plate that appeared on the next day. Diluted cell samples were then incubated in the 80°C water bath for 20 min and plated on the DS agar media again to determine the number of heat-resistant spores. Colony Forming Units (CFU) were counted for both total cells and heat-resistant spores. Sporulation efficiency was calculated as the percentage of heat-resistant spores versus total cells.

### Assays of β-Galactosidase Activities

Cells were cultured in MSgg (or LBGM) medium at 37°C in a shaking water bath. One milliliter of culture was collected at various time points and cells were spun down. Cell pellets were resuspended in 1 ml of Z buffer (40 mM NaH_2_PO_4_, 60 mM Na_2_HPO_4_, 1 mM MgSO_4_, 10 mM KCl, and 38 mM 2-mercaptoethanol) supplemented with 10 μl of 20 mg/ml freshly made lysozymes. All cell samples were incubated at 37°C for 30 min. Two hundred microliters of ONPG (*O*-Nitrophenyl-β-D-Galactopyranoside) dissolved in Z buffer was added to the solution to start the reactions. The reactions were stopped by adding 500 μl of 1 M Na_2_CO_3_ after solutions turned yellow. Samples were vortexed vigorously, briefly spun down, and applied for measurement of the OD_420_ using the Bio-Rad Smartspec 3000. The activity was calculated according to the following equation: OD_420_ × 1000/(ΔT_min_ × OD_600_).

### Microscopic Analysis

Cells were cultured in Difco Sporulation (DS) medium and grown at 37°C. One milliliter of culture was spun down and cell pellets were collected at each time point. Cell pellets were washed with PBS buffer and resuspended in a final amount of 100 μl PBS buffer. Five microliters of cell sample was spotted onto the center of the glass slide, and covered by a cover slip pre-treated with poly-lysine (Sigma). Cell samples were analyzed by Leica AF6000 Modular microsystems.

### Mass Spectrometry Analysis

For Mass Spectrometry (MS) analysis of the protein samples, total protein lysates from the WT and the *comER* mutants were prepared first. To do so, 5 ml of early stationary phase cultures (OD_600_ about 2.0) were harvested and washed with 2 ml of cold PBS buffer. Cell pellets were re-suspended in 500 μl PBS buffer supplemented with 200 μg/ml freshly made lysozymes, and incubated on ice for 30 min. The mixtures were then subject to sonication on ice for 3–5 times (15–20 pulses each, 50% duty). Cell lysates were centrifuged at 15000 × *g* for 30 min at 4°C to remove cell debris. The cleared supernatants were transferred to new cold tubes. The cleared lysates were applied to a 12% SDS-PAGE for size fractionation of the proteins. Protein bands of interests were cut from the SDS-PAGE and sent out for MS analysis. MS analysis was performed at the Taplin Mass Spectrometry Facility at Harvard Medical School.

## Results

### The Δ*comER* Mutants of both *B. cereus* and *B. subtilis* Showed a Defect in Biofilm Formation

**FIGURE 3:**
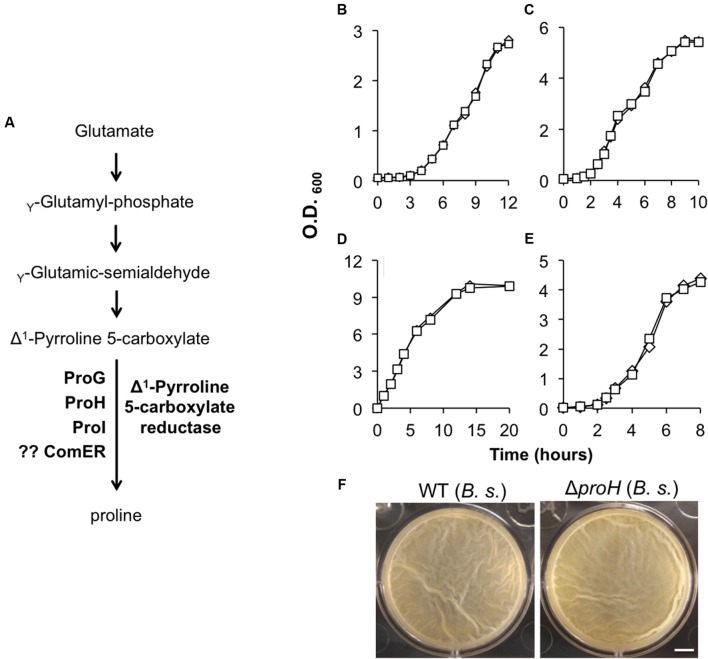
**(A)** A schematic drawing of the proposed pathway for proline biosynthesis in *B. subtilis*. ProG, ProH, and ProI resemble the Δ^1^-pyrroline-5-carboxylate reductase and were shown to be involved in the biosynthesis of proline in *B. subtilis* (Belitsky et al., 2001). No evidence shows that ComER is also involved in the last step of proline biosynthesis in *B. subtilis*. **(B,C)** Growth of the WT (diamonds) and the *comER* mutant (squares) of *B. cereus* **(B)** and of *B. subtilis* **(C)** in MSgg. Assays were repeated multiple times and representative results were shown here. **(D–E)** Growth of the WT (diamonds) and the *comER* mutant (squares) of *B. cereus* **(D)** and of *B. subtilis* **(E)** in LBGM. Assays were repeated multiple times and representative results were shown here. **(F)** Pellicle biofilm formation by the WT (3610) and the Δ*proH* deletion mutant (B268) of *B. subtilis* in LBGM. Pictures were taken after 24 h of incubation at 30°C. The scale bar represents 5 mm in length.

Genes important for biofilm formation have not been well characterized in *B. cereus*. We carried out a mini-Tn10 transposon-mediated random insertion mutagenesis in the *B. cereus* strain AR156 and screened for transposon insertion mutants with altered biofilm phenotypes (see section “Materials and Methods”). One such insertion mutant (B168) that we obtained showed an intermediate defect in pellicle biofilm formation when compared to the WT strain; after 48 h of incubation in the biofilm medium LBGM, the WT cells already developed thick floating pellicles whereas the mutant only formed a thin layer of feature-less floating mat (**Figure 2B**). We also developed a method to show the difference in pellicle biofilm robustness in a more quantitative fashion by measuring the dry weight of the floating pellicles (see section “Materials and Methods”). The result showed that this transposon mutant had an ~62% decrease in the biofilm biomass when compared to that of the WT (**Figure 2C**). The transposon insertion in this mutant was later mapped to the *comER* gene on the chromosome (indicated by the triangle; **Figure 2A**). The *comER* gene encodes a protein that resembles Δ^1^-pyrroline 5-carboxylate reductase, an enzyme involved in the last step of proline biosynthesis (**Figure 3A**). However, a loss of function mutation in *comER* does not lead to proline auxotroph in *B. subtilis* (Belitsky et al., 2001). Therefore, the function of *comER* is unclear. To further test whether the observed biofilm defect was indeed due to the insertional disruption of the *comER* gene, we complemented the Δ*comER* strain of *B. cereus* with a recombinant plasmid carrying the WT *comER* gene under a constitutive promoter (pGFP78-*comER*; see “Materials and Methods” section). The resulting complementation strain showed a WT-like biofilm phenotype and biomass (**Figures 2B,C**). In summary, our results suggested a possible role of *comER* in biofilm formation in *B. cereus*.

Since the *comER* mutant of *B. cereus* has a biofilm defective, we wondered whether the *comER* mutation in *B. subtilis* has a similar effect on biofilm formation. An insertion deletion mutation was constructed in the *comER* gene in *B. subtilis* NCIB3610 (hereafter designated as 3610; see “Materials and Methods” section). This deletion mutant (B165) and the WT strain were similarly tested for pellicle biofilm formation in LBGM. In fact, we observed a similar biofilm defect in the deletion mutant (**Figure 2D**). Interestingly, the difference in the morphology of colony biofilms between the WT and the mutant was even striking since the colony biofilm formed by the mutant was largely featureless (**Figure 2D**). Furthermore, the biofilm defect can be completely rescued by complementation of a WT copy of *comER* at an ectopic locus in the deletion mutant (**Figure 2D**). To conclude, our results indicate a significant role of *comER* in biofilm formation in both *B. cereus* and *B. subtilis*.

### Suppressor Mutations in *sinR* Rescued the Biofilm Defect Caused by Δ*comER*

**FIGURE 4:**
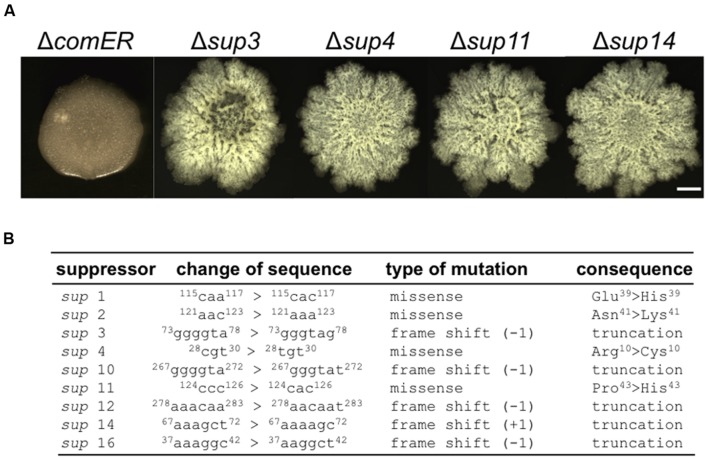
**Suppressor mutations in *sinR* rescued the biofilm defect caused by *comER*. (A)** The colony biofilm phenotype of the selected suppressor mutants of Δ*comER* (*sup*3, *sup*4, *sup*11, and *sup*14) in LBGM. The scale bar represents 5 mm in length. **(B)** A description of the characterized suppressor mutations in the nine suppressor mutants. All the putative suppressor mutations were mapped to the coding region of the *sinR* gene; Some are missense mutations while others are frame-shift mutations resulting in truncated SinR proteins.

It is interesting to note that putative suppressors of the *B. subtilis* Δ*comER* mutant occasionally arise during biofilm development (indicated by arrows in **Figure 2D**). These putative suppressors were isolated. On LBGM agar plates, we showed that the selected suppressor mutants formed much more robust colony biofilms with complex surface features than the Δ*comER* mutant (**Figure 4A**). We also tried to map the suppressor mutations in the mutants by using a candidate approach (sequencing selected genetic loci, see “Materials and Methods” section). Surprisingly, in nine out of the 11 suppressor mutants that we isolated, the suppressor mutations were all mapped to the coding region of the *sinR* gene, which include missense mutations in amino acid residues critical for SinR activities and frame-shift mutations resulting in truncated SinR proteins (**Figure 4B** and Supplementary Figure S1). Note that *sinR* encodes the biofilm master repressor for the matrix genes (Kearns et al., 2005). The above result suggests that Δ*sinR* is epistatic to Δ*comER* in the pathway controlling biofilm formation in *B. subtilis*.

### The Role of *comER* in Biofilm Formation Does Not Involve Proline Biosynthesis

The *comER* gene lies next to a three-gene operon *comEA-EB-EC* (**Figure 2A**) (Hahn et al., 1993). In previous studies, it was shown that *comEA and comEC* are important for genetic competence in *B. subtilis*, whereas *comEB* and *comER* are dispensable for that (Hahn et al., 1993; Inamine and Dubnau, 1995). Although *comER* is predicted to encode a protein that resembles Δ^1^-pyrroline 5-carboxylate reductase, there is no evidence that *comER* is needed for proline biosynthesis in *B. subtilis* (Belitsky et al., 2001). In fact, there are at least three other proteins (ProG, ProH, and ProI; **Figure 3A**) encoded by genes homologous to *comER* that were shown to collectively play important roles in proline biosynthesis in *B. subtilis* (Belitsky et al., 2001).

In *B. cereus*, those *comE* genes are highly conserved. Although the exact function of *comEA* and *comEC* has not been investigated in *B. cereus*, it has been shown that many of the competence genes whose functions are well characterized in *B. subtilis* are also highly conserved in *B. cereus* (Kovács et al., 2009). In addition, previous studies showed that *B. cereus* strains became genetically competent when they were genetically manipulated (e.g., by overexpression of the *B. subtilis* gene encoding the competence master regulator ComK; Mirończuk et al., 2008). This indicates that the genetic competence program may be present in *B. cereus* as well. To test if it is still possible that *comER* may be involved in proline biosynthesis, we compared the growth rate of AR156 and the *comER* transposon insertion mutant in *B. cereus* (B168) in a defined minimal medium (MSgg) without addition of exogenous proline. We saw no difference in growth rate between the two strains (**Figure 3B**), suggesting that in *B. cereus, comER* is also dispensable for proline biosynthesis. We also confirmed the result from the previous study that the *comER* deletion mutant of *B. subtilis* had no difference in growth rate from the WT strain when grown in the same minimal medium (**Figure 3C**) (Belitsky et al., 2001). In addition, no difference in growth rate was seen between the WT strains and the mutants in both *B. subtilis* and *B. cereus* in the biofilm medium LBGM (**Figures 3D,E**), which further ruled out the possibility that the defective biofilm phenotype of the *comER* mutants is simply due to impaired growth. Lastly, we also estimated the ratio of viable cells versus total cells in the population for both the WT strains and the *comER* mutants grown in DS medium under shaking conditions. Our results (Supplementary Figure S2) showed that most cells of both the WT strains and the *comER* mutants seemed to be alive when entering stationary growth phase. For *B. subtilis*, the ratio of live cells was at 96% for the WT and 95% for the *comER* mutant, while for *B. cereus*, the ratio was at 95% for both the WT and the Δ*comER* mutant. Thus, the ratio of the dead cells seemed to be low and had little variations between the WT and the *comER* mutant in both *B. subtilis* and *B. cereus* (Supplementary Figure S2).

On the other hand, the *proH* mutant of *B. subtilis*, which was previously shown to be deficient in proline biosynthesis (Belitsky et al., 2001), formed almost identical pellicle biofilms in LBGM to that of the WT (**Figure 3F**). Taken together, our results argue against a possible link between proline biosynthesis and the role of *comER* in biofilm formation in both *B. subtilis* and *B. cereus*.

### The Δ*comER* Mutants in both *B. cereus* and *B. subtilis* Showed Defective or Delayed Sporulation

**FIGURE 5:**
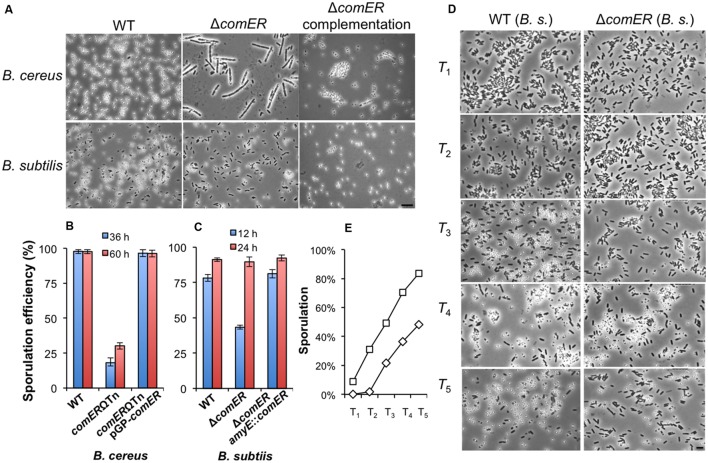
**The *comER* mutants of *B. cereus* and *B. subtilis* show defective or delayed sporulation. (A)** Phase-bright microscopic images of the spore-forming WT cells, the *comER* mutants, and the *comER* complementation strain of *B. cereus* (upper panels) and *B. subtilis* (lower panels). Cells were grown in DS medium at 37°C with shaking for 36 h for *B. cereus* or 12 h for *B. subtilis* prior to microscopic analysis. The scale bar represents 4 μm in length for all panels. **(B,C)**. Ratio of heat-resistant spores to total cells for the WT strains, the *comER* mutants, and the *comER* complementation strains of *B. cereus* **(B)** and *B. subtilis* **(C)**. Heat kill experiments were performed for cells growing in DS medium for 36 and 60 h (for *B. cereus*) or 12 and 24 h (for *B. subtilis*) and the ratio is presented as the percentage numbers. **(D)** Representative microscopic images of the WT (3610) and the *comER* mutant (B165) cells during sporulation. Cells were grown in DS medium to stationary phase and were collected on hourly basis after T_0_, which is defined as the start of the stationary phase. The scale bar shown at the right-hand corner is 3 μm in length and represents for all panels in **(D)**. **(E)** Ratio of the heat-resistant spores in the WT (squares) and the *comER* mutant cells (diamonds) of *B. subtilis*. Ratio of heat-resistant spores was calculated based on heat-kill experiments and shown as a percentage of total cells.

Our results suggest that *comER* plays a significant role in biofilm formation in both *B. cereus* and *B. subtilis*. Since regulatory pathways governing biofilm formation and sporulation overlap in these two *Bacillus* species, we were curious about whether *comER* also plays a role in sporulation. Upon further characterization, we noticed that the *comER* mutant of *B. cereus* had an alteration in the timing of sporulation (**Figure 5A**). For the WT AR156, when grown in sporulation medium (DS) at 37°C for about 36 h, nearly 100% of phase-bright spores were observed in the population (**Figure 5A**, upper-left panel), while under the same conditions, the *comER* mutant showed lots of short chains with phase-bright endospores seen in only about a quarter of the total cells (**Figure 5A**, upper-middle panel). Heat kill experiments were performed to compare the ratio of heat-resistant spores between the WT and the *comER* mutant of *B. cereus*. The result was consistent with the observation under microscope, showing that the WT cells contained about 98% heat-resistant spores, while in contrast the *comER* mutant had only about 18% heat-resistant spores (**Figure 5B**). Even after prolonged incubation for about 60 h, the ratio of heat-resistant spores of the *comER* mutant still largely lagged behind that of the WT cells (**Figure 5B**). This indicates that the *comER* mutant of *B. cereus* has a defect in sporulation.

Similarly, we compared sporulation efficiency between the WT and the *comER* mutant in *B. subtilis*. This time, after 12 h of shaking growth in the DS medium, a rather milder difference was seen in the ratio of heat-resistant spores between the two strains (**Figures 5A,C**, 78% versus 43%). After prolonged incubation for about 24 h, the ratio of the heat-resistant spores of the *comER* mutant of *B. subtilis* caught up with that of the WT strain (both stands at about 90%, **Figure 5C**). In both *B. subtilis* and *B. cereus*, the *comER* complementation strains showed WT-like sporulation efficiency either when observed under microscopy for the ratio of phase-bright spores or in heat-kill experiments (**Figures 5A–C**).

The results of the sporulation assay from the *comER* mutant of *B. subtilis* indicated that there might be a delay in the timing of spore formation in the *comER* mutant (**Figure 5C**). We did further characterization on this by comparing the timing of the appearance of phase-bright spores between the WT and the *comER* mutant (**Figure 5D**). This was done in a shaking culture in DS medium for a period of 12 h. Every hour after T_0_, cell samples for both the WT strain and the *comER* mutant were collected and analyzed by microscopy. Representative images were shown in **Figure 5D**. The ratio of heat-resistant spores were similarly assayed and summarized in **Figure 5E**. These results suggest that for *B. subtilis*, there seems to be a delay (rather than a defect seen in *B. cereus*) in the sporulation process in the *comER* mutant when compared to the WT strain (estimated to be about 2 h). For instance, the ratio of the phase-bright spores in the T_3_ sample in the WT was similar to that in the T_5_ sample in the *comER* mutant (**Figure 5E**). Therefore, in addition to its role in biofilm formation, *comER* also seems to play a role in sporulation in both *B. subtilis* and *B. cereus*. A previous report also investigated the possible role of *comER* in sporulation in a domesticated *B. subtilis* strain (Belitsky et al., 2001). The authors indicated no difference in sporulation between the WT and the *comER* mutant. Since no experimental result was presented in that study (Belitsky et al., 2001), we assumed that the authors might have examined sporulation in the domesticated *B. subtilis* strain after prolonged incubation (e.g., 24 h).

### Δ*comER* Causes Lowered Spo0A~P Activities in *B. subtilis*

It is known that in *B. subtilis* both biofilm formation and sporulation depend on the same master regulator Spo0A albeit biofilm induction replies on intermediate levels of phosphorylated Spo0A (Spo0A~P) whereas initiation of the sporulation process demands high levels of Spo0A~P (Stragier and Losick, 1996; Shank and Kolter, 2011). Thus, it is possible that in the *comER* mutants, levels of Spo0A~P may decrease or Spo0A activation is somehow delayed, which in turn causes defects in both biofilm formation and sporulation in the *comER* mutants. It is also possible that in the *comER* mutant, there might be less cells expressing Spo0A~P (the so-called Spo0A~P^ON^ cells upon entry of stationary phase; Gonzalez-Pastor et al., 2003; Fujita and Losick, 2005).

**FIGURE 6:**
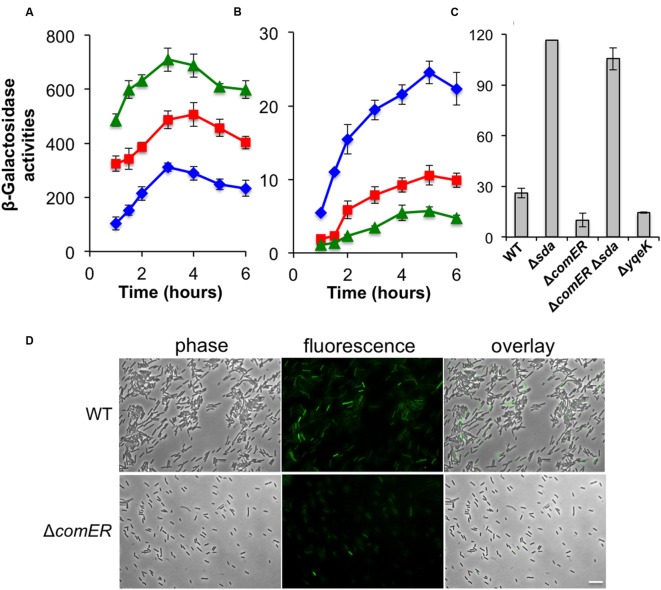
**Spo0A~P activities are reduced in the *comER* mutant of *B. subtilis*. (A)** β-Galactosidase activities of the WT strain (B223, diamonds in blue), the Δ*comER* mutant (B224, squares in red), and the Δ*spo0A* mutant (B225, diamonds in blue) that contained the P*_abrB_*-*lacZ* transcriptional fusion at the chromosomal *amyE* locus. Cells were grown in LBGM at 37°C with shaking. Cell samples were collected periodically and applied for β-galactosidase activity assays. **(B)** β-Galactosidase activities of the WT strain (YC193, diamonds in blue), the Δ*comER* mutant (B233, squares in red), and the Δ*spo0A* mutant (B234, diamonds in blue) that contained the *sdpABC*-*lacZ* transcriptional fusion on the chromosome. **(C)** Assays of β-galactosidase activities of the WT and various single and double mutants bearing the P*_epsA_*-*lacZ* reporter at the chromosomal *amyE* locus. Cells were grown in LBGM under shaking conditions to early stationary phase (OD_600_ = 2) prior to harvest. Strains used in this assay include YC1000 (WT), YL16(Δ*sda*), YL17(Δ*comER*), YL18(Δ*comER*Δ*sda*), and YL19(Δ*yqeK*). **(D)** *B. subtilis* WT strain (YY288) and the *comER* mutant (YY289) bearing the *sdpC-gfp* reporter were grown in DS medium to early stationary phase (OD_600_ = 2.0). Cells were harvested and analyzed under fluorescent microscopy. The ratio of WT cells and the *comER* mutant expressing the *sdpC-gfp* reporter was estimated to be 5 and 23%, respectively. The scale bar represents 4 μm in length.

To test the first hypothesis, we compared Spo0A~P activities in the WT and the *comER* mutant of *B. subtilis* by applying two transcriptional reporters, one for the *abrB* gene (P*_abrB_*-*lacZ*) and the other for the *sdpABC* operon (*sdpABC*-*lacZ*). The *abrB* gene is known to be under the direct negative control of Spo0A~P (Greene and Spiegelman, 1996). Thus, activities of the P*_abrB_*-*lacZ* reporter anti-correlate with Spo0A~P activities in the cells. *sdpABC* encodes a cannibalism toxin, and is under the positive regulation of Spo0A (Gonzalez-Pastor et al., 2003). We introduced the P*_abrB_*-*lacZ* and the *sdpABC*-*lacZ* reporters, respectively, into the WT and the *comER* mutant as well as the Δ*spo0A* mutant of *B. subtilis*. We then compared the β-galactosidase activities of the WT, the Δ*comER*, and the Δ*spo0A* mutant cells containing each of the reporters during shaking growth in LBGM. As shown in **Figure 6A**, activities of the *spo0A* mutant bearing the P*_abrB_*-*lacZ* reporter were consistently higher than those of the WT cells (**Figure 6A**, triangles in green for Δ*spo0A* and diamonds in blue for WT). The activities of the Δ*comER* mutant bearing P*_abrB_*-*lacZ* fell in between the WT and the Δ*spo0A* mutant (squares in red for Δ*comER*, **Figure 6A**). For the strains bearing the *sdpABC*-*lacZ* reporter, it was the opposite; the activity was significantly higher in the WT than in the Δ*spo0A* mutant (**Figure 6B**, diamonds in blue for WT and triangles in green for Δ*spo0A*). Again, the activities of the Δ*comER* mutant bearing the reporter were in between the WT and the Δ*spo0A* mutant (squares in red, **Figure 6B**). Taken together, our results suggest that at least at the level of the whole cell population, Spo0A~P activities seem to be lower in the *comER* mutant.

To further test the possibility that in the cell population of the *comER* mutant, there might be less Spo0A~P^ON^ cells when entering stationary phase, we decided to examine Spo0A~P activities in individual cells by using a *sdpC*-*gfp* fluorescent reporter whose expression is positively controlled by Spo0A~P (Gonzalez-Pastor et al., 2003; Ellermeier et al., 2006). WT *B. subtilis* cells and the *comER* mutant bearing the *sdpC-gfp* reporter were grown in LBGM to early stationary phase (OD_600_ = 2) and cells were analyzed under fluorescent microscopy for expression of the reporter. As shown in **Figure 6D**, in the cell population of the *comER* mutant, there seemed to be less cells expressing *sdpC-gfp* when compared to that of the WT cells (5% in the Δ*comER* mutant vs. 23% in the WT). Thus, our evidence suggests that in the *comER* mutant, either the activation of the Spo0A proteins or expression of the *spo0A* gene is reduced, which leads to a decreased number of Spo0A~P^ON^ cells at the onset of the stationary phase.

### Δ*sda* Is Epistatic to Δ*comER* in Regulating Biofilm Formation in *B. subtilis*

**FIGURE 7:**
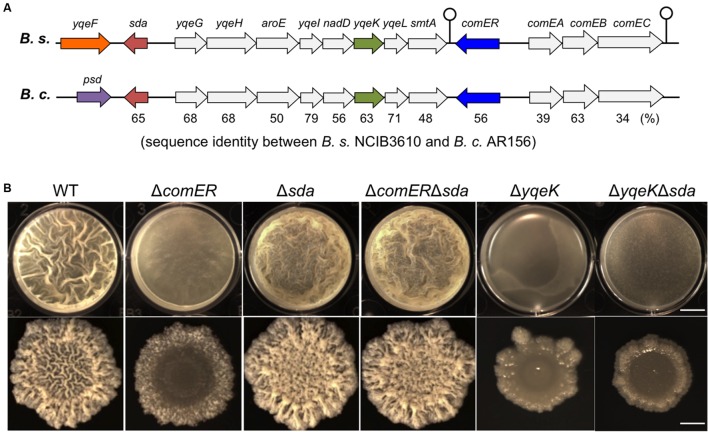
**Δ*sda* is epistatic to Δ*comER* in the control of biofilm formation in *B. subtilis*. (A)** A schematic drawing of the gene clusters on the chromosomes of *B. subtilis* 3610 and *B. cereus* AR156, in which the *comER* genes are located. The region spanning from *sda* to *comEC* is highly conserved in both *B. subtilis* and *B. cereus* in the DNA sequence as well as the genetic arrangement of the genes. The gene cluster from *yqeG* to *smtA* is predicted to form an operon (Branda et al., 2004). Sequence identities between the homologous genes are provided as percentage numbers below the genes and were analyzed by using the program ClustalW2 (http://www.ebi.ac.uk/Tools/msa/clustalw2/) and predicted protein sequences from the NCBI database. **(B)** An epitasis analysis among Δ*comER*, Δ*sda*, and Δ*yqeK*. Pellicle (B) and colony (C) biofilm formation in LBGM by the WT and various single and double mutants of *B. subtilis* was compared. Strains used in this assay include 3610 (WT), B165(Δ*comER*), B264(Δ*yqeK*), B265(Δ*sda*), B280(Δ*comER* Δ*sda*), and B281(Δ*yqeK* Δ*sda*). The scale bars in the upper and lower panels represent 4 mm and 3 mm in length, respectively.

While analyzing the nucleotide sequences flanking the *comER* gene on the chromosomes in both *B. subtilis* 3610 and *B. cereus* AR156, we noticed that the chromosomal region (of about 10-kb in length) spanning from the *comEC* gene to the *sda* gene is not only highly conserved in both strains but also has an identical arrangement of the genes (**Figure 7A**). Among the genes in that region, an eight-gene cluster (from *yqeG* to *smtA*) was previously predicted to be an operon, yet the function of the operon was not known (Branda et al., 2004). In that study, it was also shown that an insertion deletion in one of the genes, *yqeK*, resulted in a defective biofilm phenotype, suggesting that *yqeK* plays a role in biofilm formation in *B. subtilis* (Branda et al., 2004). The biofilm defect caused by Δ*yqeK* was not due to polar effect on the downstream *yqeL* and *smtA* genes (Branda et al., 2004). *yqeK* resembles genes that encode putative phosphohydrolases (Branda et al., 2004). Exactly how *yqeK* is involved in biofilm formation in *B. subtilis* is unclear. We confirmed that the *yqeK* mutant has a severe biofilm defect in LBGM too [MSgg medium was used in the previous study, (Branda et al., 2004)] (**Figure 7B**). We also showed that expression of the matrix genes is significantly down-regulated in the Δ*yqeK* mutant (**Figure 6C**).

Another interesting gene in that conserved region is *sda* (**Figure 7A**). *sda* encodes a small checkpoint protein (with the molecular weight about 6 kDa) for the control of sporulation in *B. subtilis* (Burkholder et al., 2001; Whitten et al., 2007; Veening et al., 2009). Sda negatively regulates Spo0A activity by blocking the phospho-transfer from the sensory histidine kinase A (KinA) to Spo0F (**Figure 1**) (Whitten et al., 2007). An *sda* overexpression strain showed a strong defect in sporulation due to lowered Spo0A activities, whereas the *sda* null mutation promoted sporulation even under less favorable conditions (such as in LB medium; Burkholder et al., 2001; Hoover et al., 2010). Although it may seem obvious, no investigation has been reported on the role of *sda* in biofilm formation. We constructed an *sda* null mutation in *B. subtilis* and tested the biofilm phenotype of the mutant. As shown in **Figure 7B**, in LBGM, the *sda* mutant formed equally robust pellicle and colony biofilms when compared to the WT strain. The difference in biofilm robustness between the WT and the Δ*sda* mutant was much clear on non-biofilm media (e.g., LB agar, Supplementary Figure S3), a feature that is frequently seen in hyper-robust biofilm mutants such as Δ*sinR* (Chai et al., 2010; Subramaniam et al., 2013). Our results suggest that Sda is also involved in the control of biofilm formation in *B. subtilis*.

Since Sda is known to block phospho-relay and therefore Spo0A activation, and since both *sda* and *comER* are clustered in the conserved region on the chromosomes of both *B. subtilis* and *B. cereus*, we wondered whether lowered activities of Spo0A~P we saw in the Δ*comER* mutant has anything to do with altered *sda* activities. To test our hypothesis, we first performed a simple epistasis test. We made a double mutant of Δ*sda*Δ*comER* in *B. subtilis* and compared the biofilm phenotype of the double mutant to that of the single mutants of Δ*comER* and Δ*sda*. Interestingly, the biofilm phenotype of the double mutant of Δ*comER*Δ*sda* is almost identical to that of Δ*sda*, both showing robust pellicle and colony biofilm formation (**Figure 7B**). In addition, the colony morphology on the non-biofilm LB agar plates from the double mutant also very closely resembled that of the Δ*sda* (Supplementary Figure S3). Therefore *sda* is epistatic to *comER* in the pathway regulating biofilm formation in *B. subtilis.* Interestingly, *sda* does not seem to be epistatic to *yqeK* since the biofilm phenotype of the Δ*sda*Δ*yqeK* double mutant resembled that of Δ*yqeK*, but not Δ*sda* (**Figure 7B**).

In addition to comparing the biofilm phenotype among the various single and double mutants shown above, we also measured expression of the matrix genes in those mutants. To do so, we introduced a transcriptional reporter (P*_epsA_*-*lacZ*) into various mutants, which allows us to measure the expression of the *epsA-O* operon in those mutants. We then conducted β-galactosidase assays for cells collected from pellicle biofilms. Our results suggest that the *epsA-O* operon is expressed at different levels in those mutants, much higher in the Δ*sda* single and the Δ*comER*Δ*sda* double mutant, but lower in the Δ*comER* and Δ*yqeK* single mutants, when compared to that in the WT cells (**Figure 6C**). The results from the β-galactosidase assays in general matched the observed biofilm phenotypes of the mutants. Taken together, we propose a working model, in which Sda mediates the effect of *comER* on Spo0A activities in *B. subtilis* (**Figure 1**).

It is worth pointing out that both the Δ*yqeK* and the Δ*comER* mutants show a severe biofilm defect and that in both mutants, expression of the matrix genes is much lower (**Figures 6C and 7B**), however, only the defect caused by Δ*comER* (but not by Δ*yqeK*) was rescued by Δ*sda* (**Figure 7B**). This implies that the products of the *comER* and *yqeK* genes regulate biofilm formation and matrix gene expression through different mechanisms.

### Δ*comER* Does Not Materially Alter *sda* Expression

To further explore the idea that the *comER* and *sda* genes lie in the same pathway for the regulation of biofilm formation, and that *sda* is epistatic to *comER*, we decided to test possible regulation of *sda* by *comER* by comparing expression of *sda* between the *comER* mutant and the WT strain using real-time quantitative PCR (qPCR). To do so, two pairs of primers, one for the *sda* gene in *B. subtilis* and the other for the homologous gene in *B. cereus* were used in the qPCR test (Supplementary Table S2). Our result showed that the *sda* gene was expressed at similar levels in both the WT and the *comER* mutant (Supplementary Figure S4). This is true in both *B. subtilis* and *B. cereus*, indicating that the *comER* mutation does not materially alter *sda* expression (Supplementary Figure S4). Nevertheless, it is still possible that *comER* instead plays a role in regulating Sda protein abundance or Sda activities. This can be tested in future studies with specific biochemical approaches. Based on our current evidence, we conclude that Δ*comER* does not materially alter *sda* expression.

### Δ*comER* May Reduce Levels of Spo0F in both *B. subtilis* and *B. cereus*

**FIGURE 8:**
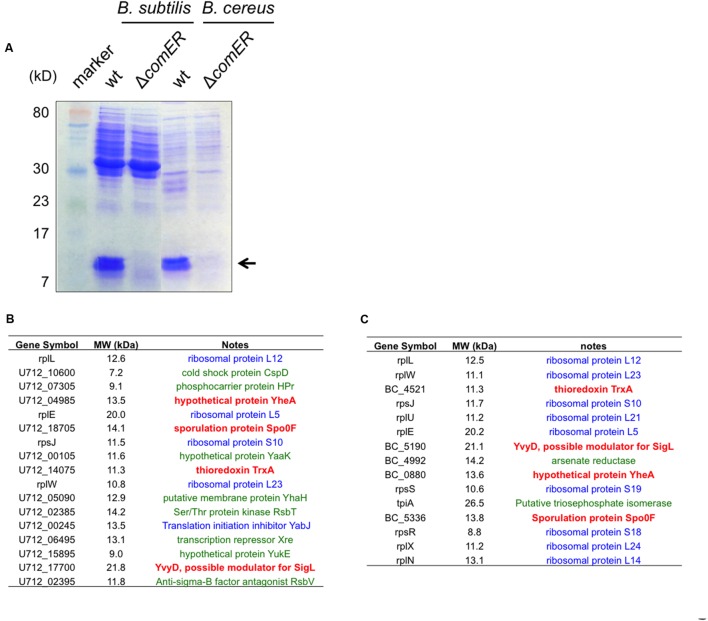
**Candidate proteins that are differentially accumulated in the WT cells and the Δ*comER* mutants of *B. subtilis* and *B. cereus*. (A)** SDS-PAGE of the total protein lysates prepared from the WT strains and the Δ*comER* mutants of *B. subtilis* and *B. cereus*. Cells were grown under shaking conditions in LBGM to early stationary phase (OD_600_ = 2). Major protein bands abundant in the lanes corresponding to the total lysates from the WT strains of *B. subtilis* and *B. cereus*, but largely absent in those from the *comER* mutants are indicated by the arrow. The size of the indicated proteins is estimated to be around 10–15 kD. **(B–C)** A list of the candidate proteins from samples of *B. subtilis* 3610 **(B)** and from *B. cereus* AR156 **(C)** based on MS analysis. Candidate proteins in blue represent ribosome or ribosome-associated proteins. Candidate proteins in green represent proteins that are present in both the WT samples and samples from the *comER* mutant (at lower levels). Candidate proteins (TrxA, YvyD, YheA, and Spo0F) in bold red are uniquely and also highly (relative counts above 10) present in the WT samples from both *B. subtilis* and *B. cereus*, but not in the samples from the *comER* mutants (Supplementary Tables S3–S6). Gene symbols were adopted from the NCBI database.

We performed a SDS-PAGE using cleared protein lysates prepared from the WT and the *comER* mutant cells. Interestingly, in both *B. subtilis* and *B. cereus*, there were significant differences between the total protein lysates prepared from the WT and from the *comER* mutant; e.g., several prominent protein bands (the size of both is estimated to be around 10–15 kD, indicated by the arrow in **Figure 8A**) shown in both of the WT samples were largely missing from the samples prepared from the two *comER* mutants. This result suggests that the *comER* mutation caused substantially lowered accumulation of at least some small, unknown proteins in both *B. subtilis* and *B. cereus*. To further characterize these small proteins, we obtained the protein bands from the lanes corresponding to the WT samples as well as the ones for the *comER* mutants (used as controls) and applied them for mass spectrometry (MS) analysis (see “Materials and Methods” section).

The results from MS analysis revealed a list of candidate proteins that were abundant in the WT samples but were substantially less in samples from the *comER* mutants. Surprisingly, among those candidate proteins, more than half of them are ribosomal proteins (labeled blue in **Figures 8B,C** and Supplementary Tables S3–S6), which seem to be common constituents in similar studies (personal communications, Godoy V, Northeastern University). Some of the candidate proteins are also present in the *comER* mutant samples albeit at lower levels (labeled green in **Figures 8B,C** and Supplementary Tables S3–S6). If excluding the above two categories of proteins, the remaining valid candidate proteins include Spo0F, YvyD, the thioredoxin TrxA, and a hypothetical protein YheA (highlighted in red; **Figures 8B,C**). YvyD is a protein of 189 aa and a potential modulator for the sigma factor SigL and ribosome dynamics (Drzewiecki et al., 1998; Tagami et al., 2012), however, no published study reported a role of YvyD or SigL in biofilm formation. In fact, we have evidence that *sigL* is not important for biofilm formation in *B. subtilis* (unpublished data). The function of *yheA* in *B. subtilis* is unknown. Thioredoxin A (TrxA) is involved in maintaining the thiol redox state and has been shown to be important in redox homeostasis, oxidative stress, sulfur metabolism, and cellular differentiation in *B. subtilis* (Smits et al., 2005).

Spo0F is a small protein of 124 amino acids (about 13 kD in molecular weight) and is well known as a key phosphor-transfer protein in the phosphor-relay that leads to protein phosphorylation and activation of Spo0A (**Figure 1**) (Stragier and Losick, 1996; Piggot and Hilbert, 2004). The *spo0F* mutant was shown to have a strong defect in both biofilm formation and sporulation (Piggot and Hilbert, 2004; Shemesh and Chai, 2013). As our data suggested, if the *comER* mutation causes reduced accumulation of Spo0F in both *B. subtilis* and *B. cereus*, this may well explain the biofilm and sporulation phenotypes of the *comER* mutants. We do hope to point out that in the SDS-PAGE (**Figure 8A**), the protein bands that seem abundant in the WT samples, but largely missing in the *comER* mutant samples consisted of many of the ribosomal proteins shown in the list (**Figures 8B,C** and Supplementary Tables S3–S6). Why they are more abundant in the WT samples than in the samples from the *comER* mutants is unclear to us. One possibility could be due to YvyD, a protein that is more abundantly present in the WT than in the *comER* mutant as we showed above (**Figures 8B,C**). YvyD was recently shown to be involved in promoting ribosome dimerization (Tagami et al., 2012), which may explain altered ribosomal protein profile in the *comER* mutant that we observed (**Figure 8**). In future studies, it will be important to apply other methods such as western immunoblot to confirm that levels of the Spo0F proteins differ significantly between the WT cells and the *comER* mutants. It is also important to verify whether some other candidate proteins in the list such as TrxA may also contribute to the role of *comER* in biofilm formation and sporulation.

## Discussion

The role of the *comER* gene in the *Bacillus* species was not identified in previous studies (Inamine and Dubnau, 1995; Belitsky et al., 2001). In those previous studies, highly domesticated laboratory strains of *B. subtilis* were used. Those domesticated strains are now known to be poor in the ability of forming robust biofilms (Branda et al., 2001; McLoon et al., 2011a). Our investigations carried out in the undomesticated strains of *B. subtilis* (NCIB3610) and *B. cereus* (AR156) show that the *comER* gene plays an important role in the regulation of biofilm formation and sporulation in both *B. subtilis* and *B. cereus*. Results from our study further suggest that *comER* may be part of the regulatory pathway that controls activation of Spo0A, the master regulator essential for both biofilm formation and sporulation. We propose that ComER may regulate Spo0A activities through its effect on the small checkpoint protein Sda (**Figure 1**). Sda is known to down-regulate Spo0A activities by blocking the phospho-transfer from the histidine kinase A to Spo0F (Whitten et al., 2007). In *B. subtilis*, the important role of Sda in sporulation as a checkpoint mechanism was already shown previously (Hoover et al., 2010). It may seem obvious that *sda* is likely involved in biofilm formation as well due to its strong regulation on Spo0A, but nevertheless it was not shown. In this study, we demonstrated that this checkpoint protein also plays an important role in the transition from free-living motile cells to sessile, biofilm-forming cells. Taken together, our results suggest a broader role of the Sda protein during decision-making for alternative cell fates (planktonic growth, biofilm, sporulation, etc.) in *B. subtilis.*

The regulation of Sda activities has been investigated previously and was shown to occur at different levels (Veening et al., 2009; Hoover et al., 2010). At the transcriptional level, *sda* is primarily regulated by the replication initiation protein DnaA, in response to cellular physiological conditions (**Figure 1**) (Veening et al., 2009; Hoover et al., 2010). When cells are in rapid growing mode, levels of the DnaA proteins are relatively high, which activate expression of *sda*. Sda in turn effectively blocks Spo0A activation and entry of spore development. Thus, Sda acts as a checkpoint protein to prevent cells from entering sporulation prematurely. This can be reversed when cellular physiological conditions and DnaA activities change (Veening et al., 2009; Hoover et al., 2010). Sda proteins are also regulated at the post-translational level by proteolysis (Ruvolo et al., 2006). During the initiation of sporulation in *B. subtilis*, a proteolysis mechanism triggers degradation of Sda by ClpXP and subsequently allows Spo0A activation (Ruvolo et al., 2006). In this study, we postulate that Sda may be regulated by another mechanism at the post-translational level, even though the details are still unclear. In particular, we speculate that ComER may regulate the activities of Sda, instead of the gene expression of *sda* or Sda protein production since our results did not support that idea that the *comER* mutation may cause either altered expression of *sda* or altered production of the Sda proteins. Based on structural predictions (HHPred^1^), ComER most strongly resembles Δ^1^-pyrroline-5-carboxylate reductases (100% probability) and prephenate dehydrogenases (99.6% probability) from various sources (FY, personal observations), indicating that ComER is possibly an oxidoreductase for a small metabolite. In future studies, it will be interesting to further understand how ComER regulates Sda activities.

In this study, we also observed that the protein levels of Spo0F, an important phospho-transfer protein for mediating activation of Spo0A by Sda, were reduced in the *comER* mutant. Apparently, altered activities of Sda (presumably caused by Δ*comER*) alone are not sufficient to explain this result since the primary activity of Sda is to block phospho-transfer from Kin histidine kinases to Spo0F. However, it is known that genes for the intermediate phospho-relay proteins (Spo0F and Spo0B) and Spo0A are under the control of a feedback regulation (Chastanet et al., 2010). Lowered levels of Spo0A should further decrease the expression of *spo0F* indirectly through the effect of Spo0A on the sigma factor H, which is required for expression of *spo0F* as well as other genes whose products are involved in phospho-relay (Predich et al., 1992). Therefore, lowered Spo0F levels could be due to lowered activities of Spo0A and the feedback mechanism. In summary, our studies suggest that the small checkpoint protein Sda may have a broader role in the cell development processes in the *Bacillus* species.

## Author Contributions

FY, J-hG, and YC designed the experiments. FY, YY, LW, and YL performed the experiments. FY, J-hG, and YC analyzed the results and wrote the manuscript.

## Conflict of Interest Statement

The authors declare that the research was conducted in the absence of any commercial or financial relationships that could be construed as a potential conflict of interest.

## Footnotes

1 http://toolkit.tuebingen.mpg.de/hhpred
