# Supplementary material for: The comER Gene Plays an Important Role in Biofilm Formation and Sporulation in both Bacillus subtilis and Bacillus cereus
Source: Front Microbiol. 2016 Jun 28;7:1025. doi: 10.3389/fmicb.2016.01025 (PMC4923064; doi:10.3389/fmicb.2016.01025)
Supplement: Supplementary file 1 [file Data_Sheet_1.PDF]

```
                                                    tgt(S4)      aag gct(S16)
  1 - ttg att ggc cag cgt att aaa caa tac cgt aaa gaa aaa ggc tac tca cta tca gaa cta
       M   I   G   Q   R   I   K   Q   Y   R   K   E   K   G   Y   S   L   S   E   L

              aaa agc(S14)
                      ggg tag (S3)                                            cac(S1)
 61 - gct gaa aaa gct ggg gta gcg aag tct tat tta agc tca ata gaa cga aat cta caa acg
       A   E   K   A   G   V   A   K   S   Y   L   S   S   I   E   R   N   L   Q   T

      aaa(S2)
          cac(S11)
121 - aac ccc tcc att caa ttt ctc gaa aaa gtc tcc gct gtt ctg gac gtc tcg gtt cat act
       N   P   S   I   Q   F   L   E   K   V   S   A   V   L   D   V   S   V   H   T

181 - ttg ctc gat gag aaa cat gaa acc gaa tac gat ggt caa tta gat agt gaa tgg gag aaa
       L   L   D   E   K   H   E   T   E   Y   D   G   Q   L   D   S   E   W   E   K

                                   ggg tat(S10)
                                               aac aat (S12)
241 - ttg gtt cgc gat gcg atg aca tcc ggg gta tcg aaa aaa caa ttt cgt gaa ttt tta gat
       L   V   R   D   A   M   T   S   G   V   S   K   K   Q   F   R   E   F   L   D

301 - tat caa aaa tgg aga aaa tcc caa aaa gag gag tag
       Y   Q   K   W   R   K   S   Q   K   E   E   •
```

Figure S1

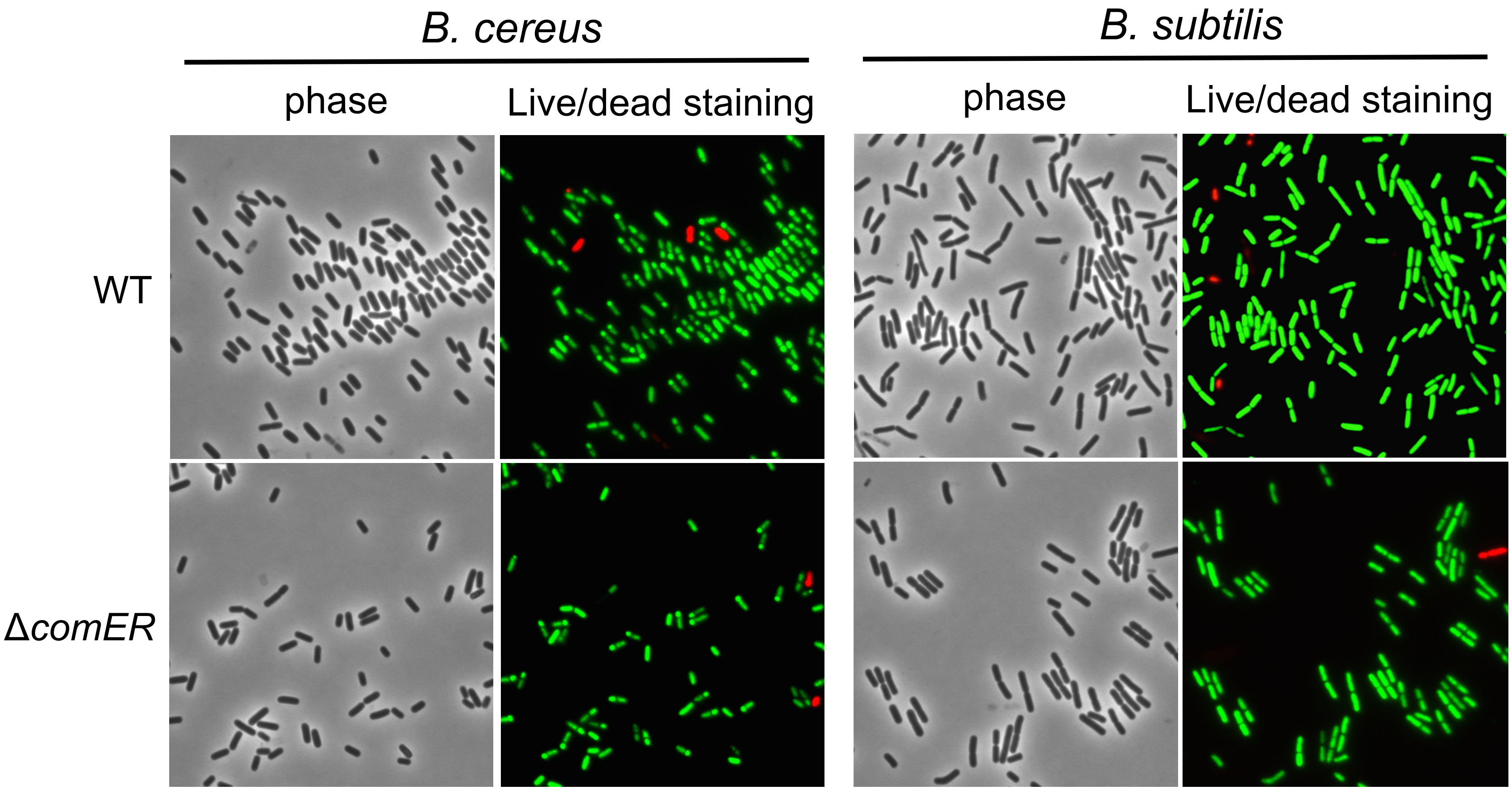

B. cereus
B. subtilis
phase
Live/dead staining
phase
Live/dead staining
WT
ΔcomER

Figure S2

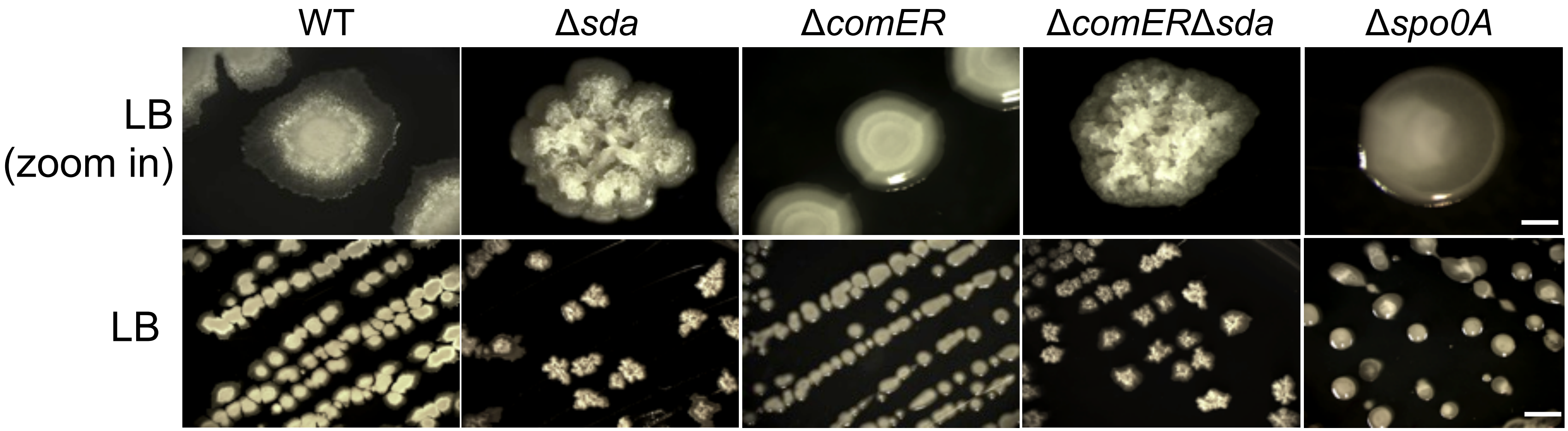

Figure S3

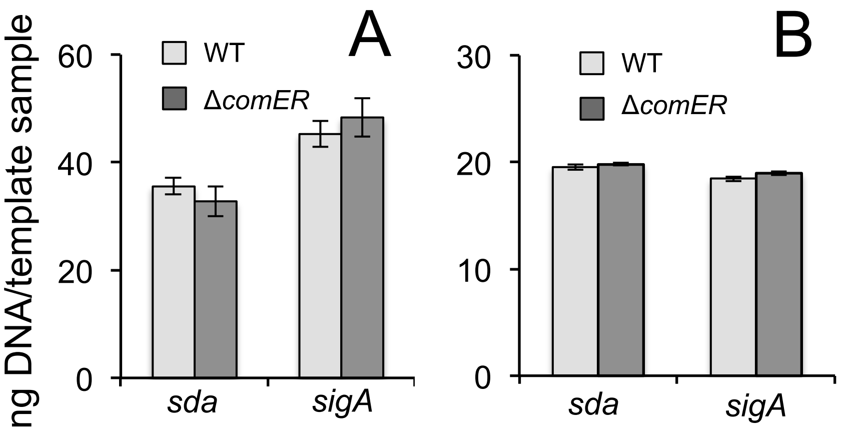

ng DNA/template sample
A
60
40
20
0
WT
ΔcomER
sda
sigA
B
30
20
10
0
WT
ΔcomER
sda
sigA

Figure S4

Supplemental figure legends

Fig. S1. A summary of mutations found in the coding region of *sinR* in the suppressor mutants. Shown are the DNA sequences of the coding region of the *sinR* gene in *B. subtilis* 3610. Mutations in the *sinR* gene in each of the 9 selected suppressor mutants of Δ*comER* were identified by DNA sequencing. The strain names of the associated suppressor mutants are indicated in the parenthesis.

Fig. S2. Live/dead cell staining of the wild type strains and the Δ*comER* mutants of *B. subtilis* and *B. cereus*. Wild type strains and the *comER* mutants of *B. subtilis* (3610 and B165, respectively) and of *B. cereus* (AR156 and B168, respectively) were grown in DS media to early stationary phase ($O.D._{600}$=2.0). Cells were harvested and treated with dyes for live/dead cell staining and examined under phase-contrast and fluorescent microscopy. Live cells were stained in green whereas dead cells were in red.

Fig. S3. Colony morphology of the wild type strain (3610), and Δ*comER* deletion mutant (B165), the Δ*sda* deletion mutant (B265), the Δ*comER*Δ*sda* double mutant (B280), and the Δ*spo0A* mutant (RL4620) of *B. subtilis* on LB agar plates. Upper panels are zoom-in images from the lower panels. The scale bar in upper panels represents 0.4 mm in length; the bar in lower panels represents 4 mm in length.

Fig. S4. Δ*comER* does not significantly alter *sda* expression or production of Sda proteins. (A). Results from the qPCR experiments to compare the abundance of the *sda* transcripts from the wild type (3610) and the *comER* mutant (B165) of *B. subtilis*. The *sigA* gene (encoding the house-keeping sigma factor A) of *B. subtilis* was used as an internal control. **(B).** Similar results from the qPCR experiments to compare the abundance of the *sda* transcripts from the wild type (AR156) and the *comER* mutant (B168) of *B. cereus*. The *sigA* gene of *B. cereus* was used as an internal control. Known concentrations of genomic DNA were used to generate the standard curve. The y-axis values represent the amount of DNA per template sample.

Supplemental Methods

Real-time RT-PCR. Cells were grown to mid-log phase in LB medium and harvested. Total RNAs were isolated from the cells by using the Easy RNA Miniprep kit (Qiagen) according to the manufacturer's instructions. 1 ng of total RNA was applied to a reverse transcription reaction using AMV First Strand cDNA Synthesis Kit as described in the provided protocol (New England Biolabs). Real time PCR was performed to quantify the level of expression of the *sda* gene with the KAPA SYBR fast qPCR kit (KAPA Biosystems) according to the manufacturer's instructions. The resulting cDNA samples were serially diluted and used as the template for real time PCR. In parallel, purified genomic DNAs with known concentrations were used to generate a standard curve. PCR was performed in the Mastercycler ep realplex by using the following programs: one cycle of 95°C for 3min, 40 cycles of 95°C for 3 s, 53°C for 20 s, and 60°C for 20 s. All quantitative PCR were analyzed using the Mastercycler ep realplex software.

Live/dead cell staining. Live/dead cell staining followed the protocol provided by the manufacture (Invitrogen, USA). In brief, *B. subtilis* and *B. cereus* cells were grown in DS medium in shaking culture to early stationary phase ($O.D._{600}$=2.0). Cells were collected and washed with PBS buffer twice. Cells were resuspended and diluted to about $5x10^7$ cells per ml in PBS buffer. 1-µl of reconstituted fluorescent reactive dye was added to 1-ml of the cell suspension and mixed well. Cell samples were incubated on ice for 30 min and were concentrated for examination by fluorescent microscopy.

Table S1: Strains used in this study

Strain or plasmid	Genotype	Reference
3610	An undomesticated strain capable of forming robust biofilms	(Branda et al., 2001)
PY79	A laboratory strain for genetic manipulations	(Branda et al., 2001)
AR156	An environmental isolate of *B. cereus*	(Niu et al., 2011)
BKE25630	Δ*yqeK* in *B. subtilis*168, Mls^R	BGSC
BKE25680	Δ*sda* in *B. subtilis* 168, Mls^R	BGSC
DS1010	A *B. subtilis* strain with the plasmid pIC333 Spc^R, Mls^R	(Kearns et al., 2004)
EG443	*sdp*Ω*sdpABC-gfp*::*spec*, Spc^R in PY79	(Gonzalez-Pastor et al., 2003)
FC287	*amyE::*P_{abrB}*-lacZ::spec* in 3610	(Chu et al., 2008)
RL1941	An *E. coli* strain with the plasmid pDG1515 Tc^R	Losick lab
RL2559	Δ*proH* in PY79, Spc^R	(Eichenberger et al., 2003)
RL3545	An *E. coli* strain with the plasmid pAH52, Mls^R	Losick lab
RL3555	*sdp*Ω*sdpABC-lacZ*::*spec*, Spc^R in PY79	(Fujita et al., 2005)
RL4620	Δ*spo0A*::*kan* in 3610, Kan^R	(Chu et al., 2008)
YC193	*sdp*Ω*sdpABC-lacZ*::*spec*, Spc^R in 3610	This study
YC1000	*amyE*::P_{epsA}*-lacZ*::cm, Cm^R in 3610	(Subramaniam et al., 2013)
B79	AR156 strain with the plasmid pIC333	This study
B165	Δ*comER* in 3610, mls^R	This study
B168	Transposon insertion in *comER* in AR156, Spc^R	This study
B223	*amyE*:: P_{abrB} *-lacZ* in 3610, Spc^R	This study
B224	Δ*comER*, *amyE*:: P_{abrB}*-lacZ* in 3610, Mls^R Spc^R	This study
B225	Δ*spo0A::kan*, *amyE*:: P_{abrB}*-lacZ* in 3610, Kan^R Spc^R	This study
B233	Δ*comER::tc sdp*Ω*sdpABC-lacZ*::*spec*, Tc^R, Spc^R in 3610	This study
B234	Δ*spo0A::Kan sdp*Ω*sdpABC-lacZ*::*spec*, Kan^R, Spc^R in 3610	This study
B264	Δ*yqeK* in 3610, Mls^R	This study
B265	Δ*sda* in 3610, Mls^R	This study
B266	Δ*sda* in 3610, Tc^R	This study
B268	Δ*proH* in 3610, Spc^R	This study
B280	Δ*sda*; Δ*comER* in 3610, Mls^R, Tc^R	This study
B281	Δ*sda*; Δ*yqeK* in 3610, Tc^R, Mls^R	This study
YL16	Δ*sda*::*mls, amyE*::P_{epsA}*-lacZ* in 3610, Mls^R, Cm^R	This study
YL17	Δ*comER*::*mls, amyE*::P_{epsA}*-lacZ* in 3610, Mls^R, Cm^R	This study
YL18	Δ*sda*::*mls, comER*::*tet, amyE*::P_{epsA}*-lacZ* in 3610, Mls^R, Tet^R, Cm^R	This study
YL19	Δ*yqeK*::*mls, amyE*::P_{epsA}*-lacZ* in 3610, Mls^R, Cm^R	This study
YL21	*amyE*::P_{comER}*-lacZ* in 3610, Cm^R	This study
YL46	Δ*comER*, *amyE*::*comER*, Mls^R, Cm^R	This study
YY288	*sdpABC*Ω*gfp*::*spec* in 3610, Spc^R	This study
YY289	*sdpABC*Ω*gfp*::*spec* and Δ*comER*::*mls* in 3610, Spc^R, Mls^R	This study
YY298	Transposon insertion in *comER,* pGP-*comER* in AR156, Spc^R, Tet^R	This study
pAH52	A modified pBluescript plasmid as a template for long-flanking PCR mutagenesis, Amp^R, Spc^R.	(Ferguson et al., 2007)
pDG1515	A modified pBluescript plasmid as a template for long-flanking PCR mutagenesis, Amp^R, Tet^R.	(Guérout-Fleury et al., 1995)
pDG1662	An *amyE* integration vector for *B. subtilis*, Cm^R, Spc^R, Amp^R.	(Guérout-Fleury et al., 1996)
pGFP78	A shuttle plasmid capable of replicating in *E. coli* and *B. cereus*, Tet^R	(Gao et al., 2015)

Table S2: Primers used in this study.

Primer name	Primer sequence
delta-sda-P1	5'- GTAACTCCGCACCCGATACAAA -3'
delta-sda-P2	5'- CAATTCGCCCTATATTGAGTCGTGGGAG -3'
delta-sda-P3	5'- CCAGCTTTTGTTCCCTTTAGTGAGTAATA -3'
delta-sda-P4	5'- GCAGTGGGTCAATATATTAAAA -3'
delta-comER-P1	5'- GAAGAATCGCATTCATTTCAGCGTGA -3'
delta-comER-P2	5'- CAATTCGCCCTATGAGTCGTCTCTATCTTCAATGGTTTCCC -3'
delta-comER-P3	5'- CCAGCTTTTGTTCCCTTTAGTGAGCAGTTTCATGTGTAGACGAGT -3'
delta-comER-P4	5'- GGATCAAGCTGATGAAAACGGCATTC -3'
P-sda-F	5'- TGGGTTCCTAGCATGAGAAAACT -3'
P-sda-R	5'- TGTCCGAGCGATCTTCTTTT-3'
P-BC-sda-F	5'- GGAACAGTTATCTACTGAGTTAC -3'
P-BC-sda-R	5'- GACGATTTGACAAGCTTGTCC -3'
P-sinR-F	5'- AAA AGCTGGGGTAGCGAAGT -3'
P-sinR-R	5'- CGCATCGCGAACCAATTTCT -3'
sinR-F	5'- TCACAAGGAAGGTGATGAC-3'
sinR-R	5'- GTGCCTCTGCTCAGGCACTA-3'
abrB-F	5'- AAGTATCTCTTGGGAGGAGA-3'
abrB-R	5'- GTTCTTGTGTCATAACAAGA-3'
sda-F	5'- AAGGAGGTGCCTCTCCC-3'
sda-R	5'- TAGACAAATTCCTATTA-3'
P-BS-sigA-F	5'- GCCGAAGAAGAATTTGA-3'
P-BS-sigA-F	5'- GATACTGACAACAAGCC-3'
P-BC-sigA-F	5'- GCGACAATGACAACGAC -3'
P-BC-sigA-R	5'- TGGCGAATCCACCAAGT -3'
P-comER-F1	5'- GTCGAATTCCGTGCATGTTCCCCGCTTTC-3'
P-comER-R2	5'- GTCGGATCCCTACACATGAAACTGCTTTTT-3'
Bc-comER-OE-F:	5'- GTACTCTAGAAGGAGGGATGAAACATTGAACATAGGAATTATAG-3'
Bc-comER-OE-R:	5'- GTACAAGCTTaTGTGTGTTTATTGAATTG-3'
Tn10-113-98	5'- GCCGCGTTGGCCGATTC -3
Tn10-2235-2249	5'- GATATTCACGGTTTA -3'

Table S3. MS analysis of the protein samples of *B. subtilis* 3610.

Unique[b]	Total[a]	Symbol	Coverage	MW (kDa)	Notes[c]
9	73	rplL	59.63%	12.6	ribosomal protein L12
6	54	U712_10600	64.77%	7.2	cold shock protein CspD
8	26	U712_07305	57.76%	9.1	phosphocarrier protein HPr
15	23	U712_04985	81.35%	13.5	hypothetical protein YheA
17	20	rplE	67.25%	20.0	ribosomal protein L5
15	15	groEL	27.21%	57.0	heat shock protein GroEL
12	15	U712_18705	69.41%	14.1	**sporulation protein Spo0F[d]**
9	14	rpsJ	68.64%	11.5	ribosomal protein S10
12	13	U712_00105	85.86%	11.6	hypothetical protein YaaK
13	13	tuf	30.05%	43.4	elongation factor Tu
10	13	U712_14075	75.62%	11.3	thioredoxin TrxA
10	12	rplW	81.23%	10.8	ribosomal protein L23
12	12	U712_05090	72.35%	12.9	putative membrane protein YhaH
12	12	U712_02385	56.70%	14.2	Ser/Thr protein kinase RsbT
8	12	rpsB	30.26%	27.8	ribosomal protein S2
3	11	U712_00245	15.68%	13.5	Translation initiation inhibitor YabJ
6	11	U712_06495	31.50%	13.1	transcription repressor Xre
6	11	U712_15895	47.25%	9.0	hypothetical protein YukE
10	10	sucC	23.82%	41.2	succinyl-CoA synthetase
10	10	U712_17700	52.19%	21.8	**YvyD, possible modulator for SigL[d]**
10	10	atpA	18.29%	54.4	ATP synthetase subunit
9	9	U712_02395	59.26%	11.8	Anti-sigma-B factor antagonist RsbV
9	9	rpsC	32.77%	24.2	ribosomal protein S3
8	9	U712_03610	37.06%	14.6	Putative monooxygenase yetG
8	9	U712_07870	46.45%	13.4	hypothetical protein YlbA
9	9	tsf	22.74%	32.2	Elongation factor Ts
9	9	U712_11050	65.45%	9.7	non-specific DNA-binding protein HU
8	9	U712_11410	45.56%	12.9	anti-anti-sigma factor SpoIIAA
9	9	tig	21.62%	47.3	trigger factor
8	8	rpsG	30.24%	17.7	ribosomal protein S7
7	8	rplV	38.01%	12.3	ribosomal protein L22
7	8	U712_08920	33.16%	16.0	biofilm protien YmcA
8	8	U712_15220	36.92%	16.5	non-specific DNA binding protien Dps
8	8	rplI	47.64%	12.6	ribosomal protein L9
7	7	rpsE	30.04%	17.5	ribosomal protein S5
7	7	rplC	26.79%	22.5	ribosomal protein L3
7	7	U712_05110	34.54%	16.2	Hit-like protein for cell-cycle regulation
6	7	rplS	36.28%	13.6	ribosomal protein L19
7	7	U712_13465	32.21%	15.1	putative holiday junction resolvase YrrK

a. Total stands for the total counts of peptides corresponding to the designated protein. In general, more total counts correlate with higher abundance of the protein in the samples.
b. Unique stands for the number of distinct peptides corresponding to the same designated protein.
c. Candidates in blue represent ribosome or ribosome-associated proteins, possibly contaminations. Candidates in green represent proteins that are present in both the wild type samples and samples from the *comER* mutant. Candidates in black are uniquely present in the wild type sample of *B. subtilis*, but not the *comER* mutant.
d. Candidates (Spo0F and SigL) in red are uniquely but also highly (counts above 10) present in the wild type samples from both *B. subtilis* and *B. cereus* (Table S5), but not in the samples from the *comER* mutants (Tables S4 and S6).

Table S4. MS analysis for the protein samples of *B. subtilis* Δ*comER*.

Unique[b]	Total[a]	Symbol	Coverage	MW (kDa)	Notes[c]
13	13	U712_04985	86.54%	13.5	hypothetical protein YheA
9	11	U712_07305	75.38%	9.1	Phosphocarrier protein HPr
4	10	U712_10600	46.65%	7.2	cold shock protein CspD
7	8	rplL	48.24%	12.6	ribosomal protein L12
8	8	U712_02385	42.67%	14.2	Ser/Thr protein kinase RsbT
6	8	U712_07870	39.19%	13.4	hypothetical protein YlbA
6	6	U712_03610	31.76%	14.6	Putative monooxygenase yetG
6	6	U712_13465	33.42%	15.1	putative holiday junction resolvase YrrK
6	6	U712_10300	38.69%	12.7	transcription factor YodB
6	6	U712_15045	56.70%	7.7	uncharacterized protein YtwF
6	6	U712_19850	34.21%	15.8	universal stress protein YxiE
5	5	U712_00400	33.31%	13.4	dihydroneopterin aldolase FolB
5	5	U712_02875	31.83%	14.3	uncharacterized protein YdhG
5	5	U712_05110	27.68%	16.2	Hit-like protein involved in cell-cycle regulation
5	5	U712_05205	28.52%	15.1	uncharacterized protein YhfF
5	5	U712_06375	29.78%	15.4	Uncharacterized protein yjlC
5	5	U712_10260	33.54%	12.9	Uncharacterized protein yojF
5	5	U712_08920	27.90%	16	biofilm protein YmcA
5	5	U712_17710	32.67%	13.4	Flagellar protein FliT
4	4	U712_00105	27.17%	11.6	uncharacterized protein YaaK
4	4	U712_02315	38.65%	12.3	Thioredoxin-like protein ydbP
4	4	U712_05090	34.25%	12.9	putative membrane protein YhaH
4	4	rplV	25.48%	12.3	ribosomal protein L22
4	4	U712_09515	20.87%	14.8	Uncharacterized protein yneT
4	4	U712_10900	24.41%	12.8	Uncharacterized protein ypjD
4	4	U712_15895	35.12%	9	Uncharacterized protein yukE
4	4	U712_14780	25.71%	12.2	Uncharacterized protein ytxJ
4	4	U712_19835	21.82%	16.3	Uncharacterized protein yxxG
3	3	tuf	7.26%	43.4	elongation factor Tu
3	3	rplW	22.04%	10.8	ribosomal protein L23
3	3	rplQ	19.29%	13.6	ribosomal protein L17
3	3	U712_00250	22.05%	10.8	sporulation protein SpoVG
3	3	U712_02370	18.30%	12.8	mRNA interferase YdcE
3	3	U712_02395	20%	11.8	Anti-sigma-B factor antagonist RsbV
3	3	U712_04440	14.22%	16.3	transcriptional repressor PerR

a. Total stands for the total counts of peptides corresponding to the designated protein. In general, more total counts correlate with higher abundance of the protein in the samples.
b. Unique stands for the number of distinct peptides corresponding to the same designated protein.
c. Candidates in blue represent ribosome or ribosome-associated proteins, possibly contaminations. Candidates in green represent proteins that are present in both the wild type samples and samples from the *comER* mutant. Candidates in black are uniquely present in the wild type sample of *B. subtilis*, but not the *comER* mutant.

Table S5. MS analysis for the protein samples of *B. cereus* AR156.

Unique[b]	Total[a]	Symbol	Coverage	MW (kDa	Notes[c]
12	75	rplL	80.76%	12.5	ribosomal protein L12
11	25	rplW	82.33%	11.1	ribosomal protein L23
11	23	BC_4521	76.89%	11.3	thioredoxin TrxA
9	21	rpsJ	64.34%	11.7	ribosomal protein S10
8	19	rplU	63.53%	11.2	ribosomal protein L21
12	12	tuf	28.12%	42.8	Elongation Factor Tu
12	12	BC_0491	12.56%	84.7	formate acetyltransferase
10	12	rplE	40.95%	20.2	ribosomal protein L5
9	11	BC_5190	45.31%	21.1	**YvyD, possible modulator for SigL**
11	11	tig	20.18%	47.3	trigger factor
11	11	tsf	28.53%	32.5	Elongation factor Ts
11	11	fusA	13.92%	76.3	Elongation factor G
9	11	BC_4992	53.45%	14.2	arsenate reductase
11	11	BC_3970	18.7%	49.4	dihydrolipoamide dehydrogenase
8	11	BC_0880	45.71%	13.6	hypothetical protein YheA
6	11	rpsS	46.13%	10.6	ribosomal protein S19
8	10	tpiA	22.87%	26.5	Putative triosephosphate isomerase
9	10	BC_5336	48.09%	13.8	**Sporulation protein Spo0F[d]**
9	10	rpsR	81.2%	8.8	ribosomal protein S18
6	10	rplX	44.47%	11.2	ribosomal protein L24
9	10	rplN	57.54%	13.1	ribosomal protein L14
9	9	dnaK	14.95%	65.8	cheparonin DnaK
9	9	gcvT	18.01%	40.2	glycine cleavage system
9	9	sucC	18.05%	41.7	succinyl-CoA synthetase subunit
7	9	rplQ	39.88%	13.5	ribosomal protein L17
8	8	ldh	19.85%	34.8	lactate dehydrogenase
7	8	rplI	47.11%	12.5	ribosomal protein L9
8	8	BC_5255	16.19%	43.8	efflux protein
7	8	BC_3599	35.82%	15.6	succinyl-CoA synthetase subunit
8	8	rpsC	31.58%	24.3	ribosomal protein S3
5	8	rplV	33.47%	13.1	ribosomal protein L22
8	8	rplC	25.98%	22.7	ribosomal protein L3
6	8	serS	22.14%	27.3	serine-tRNA synthetase
7	7	groL	16.55%	57.4	GroEL
7	7	BC_5335	18.19%	30.7	fructose-biphosphate aldolase
7	7	BC_4983	18.67%	29	ABC transporter
6	7	BC_4980	26.52%	16	IscU-like protein
7	7	BC_4158	16.72%	35.8	2-oxoisovalerate dehydrogenase
5	7	BC_4049	39.92%	9.2	phosphocarrier protein HPr

a. Total stands for the total counts of peptides corresponding to the designated protein. In general, more total counts correlate with higher abundance of the protein in the samples.
b. Unique stands for the number of distinct peptides corresponding to the same designated protein.
c. Candidates in blue represent ribosome or ribosome-associated proteins, possibly contaminations. Candidates in green represent proteins that are present in both the wild type samples and samples from the *comER* mutant. Candidates in black are uniquely present in the wild type sample of *B. subtilis*, but not the *comER* mutant.
d. Candidates (Spo0F and SigL) in red are uniquely but also highly (counts above 8) present in the wild type samples from both *B. subtilis* (Table S3) and *B. cereus*, but not in the samples from the *comER* mutants (Tables S4 and S6).

Table S6. MS analysis for the protein samples of *B. cereus* Δ*comER*.

Unique[b]	Total[a]	Symbol	AVG	MW (kDa)	Notes[c]
8	8	rplL	55.34%	12.5	ribosomal protein L12
6	6	BC_4521	45.08%	11.3	thioredoxin TrxA
6	6	BC_0880	39.1%	13.6	hypothetical protein YheA
5	5	BC_2011	24.91%	16.7	non-specific DNA binding protein Dps
5	5	BC_1366	26.54%	14.9	SseB
4	5	rplN	25.89%	13.1	ribosomal protein L14
4	4	rplU	28.94%	11.2	ribosomal protein L21
4	4	rplQ	23.90%	13.5	ribosomal protein L17
4	4	spoVG	29.16%	10.9	sporulation protein SpoVG
3	3	rpmA	21.31%	10.5	ribosomal protein L27
3	3	BC_4049	25.73%	9.2	phosphocarrier protein HPr
3	3	BC_3599	14.49%	15.6	succinyl-CoA synthetase subunit
3	3	rplV	17.62%	13.1	ribosomal protein L22
2	2	rpsM	12.68%	13.8	ribosomal protein S13
2	2	BC_3986	15.34%	9.1	hypthetical protein YkuJ
2	2	BC_2887	10.2%	15.6	unknown cytoplasmic protein
2	2	rplI	12.52%	12.5	ribosomal protein L9
2	2	BC_5060	9.83%	16.5	unknown protein YjlC
2	2	BC_4992	10.34%	14.2	arsenate reductase
1	2	BC_4728	6.94%	11.6	molybdopterin protein MoeB
2	2	BC_4583	4.77%	37.6	glyceroldehyde-3P dehydrogenase
2	2	BC_3600	4.05%	44	proase HhoA
2	2	BC_2262	5.03%	37.8	macrolide glycosyltransferase

a. Total stands for the total counts of peptides corresponding to the designated protein. In general, more total counts correlate with higher abundance of the protein in the samples.
b. Unique stands for the number of distinct peptides corresponding to the same designated protein.
c. Candidates in blue represent ribosome or ribosome-associated proteins, possibly contaminations. Candidates in green represent proteins that are present in both the wild type samples and samples from the *comER* mutant. Candidates in black are uniquely present in the wild type sample of *B. cereus*, but not the *comER* mutant.

Supplemental references

Branda, S.S., Gonzalez-Pastor, J.E., Ben-Yehuda, S., Losick, R., and Kolter, R. (2001). Fruiting body formation by *Bacillus subtilis*. *Proc. Natl. Acad. Sci. USA* 98**,** 11621-11626.

Chu, F., Kearns, D., B. , Mcloon, A., Chai, Y., Kolter, R., and Losick, R. (2008). A novel regulatory protein governing biofilm formation in *Bacillus subtilis*. *Mol. Microbiol.* 68**,** 1117-1127.

Eichenberger, P., Jensen, S.T., Conlon, E.M., Van Ooij, C., Silvaggi, J., González-Pastor, J.-E., Fujita, M., Ben-Yehuda, S., Stragier, P., Liu, J.S., and Losick, R. (2003). The σE Regulon and the Identification of Additional Sporulation Genes in Bacillus subtilis. *Journal of Molecular Biology* 327**,** 945-972.

Ferguson, C.C., Camp, A.H., and Losick, R. (2007). gerT, a Newly Discovered Germination Gene under the Control of the Sporulation Transcription Factor σ(K) in Bacillus subtilis. *Journal of Bacteriology* 189**,** 7681-7689.

Fujita, M., Gonzalez-Pastor, J.E., and Losick, R. (2005). High- and Low-threshold genes in the Spo0A regulon of *Bacillus subtilis*. *J. Bacteriol.* 187**,** 1357-1368.

Gao, T., Foulston, L., Chai, Y., Wang, Q., and Losick, R. (2015). Alternative modes of biofilm formation by plant-associated Bacillus cereus. *MicrobiologyOpen* 4**,** 452-464.

Gonzalez-Pastor, J.E., Hobbs, E.C., and Losick, R. (2003). Cannibalism by sporulating bacteria. *Science* 301**,** 510-513.

Guérout-Fleury, A.-M., Shazand, K., Frandsen, N., and Stragier, P. (1995). Antibiotic-resistance cassettes for Bacillus subtilis. *Gene* 167**,** 335-336.

Guérout-Fleury, A.M., Frandsen, N., and Stragier, P. (1996). Plasmids for ectopic integration in *Bacillus subtilis*. *Gene* 180**,** 57-61.

Kearns, D.B., Chu, F., Rudner, R., and Losick, R. (2004). Genes governing swarming in Bacillus subtilis and evidence for a phase variation mechanism controlling surface motility. *Molecular Microbiology* 52**,** 357-369.

Niu, D.-D., Liu, H.-X., Jiang, C.-H., Wang, Y.-P., Wang, Q.-Y., Jin, H.-L., and Guo, J.-H. (2011). The Plant Growth–Promoting Rhizobacterium Bacillus cereus AR156 Induces Systemic Resistance in Arabidopsis thaliana by Simultaneously Activating Salicylate- and Jasmonate/Ethylene-Dependent Signaling Pathways. *Molecular Plant-Microbe Interactions* 24**,** 533-542.

Subramaniam, A.R., Deloughery, A., Bradshaw, N., Chen, Y., O’shea, E., Losick, R., and Chai, Y. (2013). A serine sensor for multicellularity in a bacterium. *eLife* 2.
